# Exploring the predictive performance of deep learning for fracturing fluid flowback and shale gas production

**DOI:** 10.1038/s41598-025-26761-z

**Published:** 2025-11-28

**Authors:** Shasha Sun, Wenhua Bai, Zhaoyuan Shao, Zhensheng Shi, Tianqi Zhou, Yuanjiang Yu, Jian Sun, Lisha Peng, Lei Zhang

**Affiliations:** 1 Research Institute Petroleum Exploration and Development, Beijing, 10083 China; 2 Zhejiang Oilfield Exploration and Development Research Institute, Hangzhou, 310000 China; 3 Zhejiang Oilfield Chongqing Natural Gas Business Department, Chongqing, 404100 China

**Keywords:** Shale gas production, Fracturing fluid flowback, CNN-Transformer, CNN-LSTM, Natural gas, Petrol

## Abstract

The big data system has been developed to optimally combine numerical-model predictions with actual measurements from the gas shale play to create the best estimates of current shale gas conditions and their uncertainties, improving our ability to forecast and understand the shale gas production variations. However, considering the hydraulic fracturing fluid flowback in predicting shale gas production introduces new challenges. For example, complexities in the flow of gas-water two-phase fluid defy traditional numerical simulation, while flowback are complicated by strong environmental disruptions, and shale gas production encounters substantial noise interference. Here, we developed CNN-Transformer, a production and fluid flowback predicted system using deep learning, by integrating a convolutional neural network (CNN) and a Transformer network. CNN’s receptive field focus irregular observational data on the local region correlation of the sequence, while Transformer network extracts the state information of historical production and flowback volumes from previous time frames, and predicts future time states, aiming to capture temporal patterns. We contrast the performance of CNN-Transformer, CNN-LSTM, CNN-GRU-AM models in predicting shale gas production, flowback volume and the relationship between the two. We show that the CNN-Transformer outperforms the other models, with R²=0.644 and RMSE = 0.1424, compared to CNN-GRU-AM (R²=0.6068, RMSE = 0.1513) and CNN-LSTM (R²=0.5727, RMSE = 0.1618) in predicting flowback volume. We conclude that CNN-Transformer (R^2^ = 0.72, RMSE = 0.3824) markedly reduces analysis error of the shale gas production, outperforming both CNN-LSTM (R^2^ = 0.4911, RMSE = 0.44912) and CNN-GRU-AM (R^2^ = 0.5705, RMSE = 0.4249). We deem our results to lay a foundation for further debates on striking a balance between fracturing fluid flowback volume and prediction of shale gas production.

**Supplementary Information:**

The online version contains supplementary material available at 10.1038/s41598-025-26761-z.

## Introduction

**Fig. 1:**
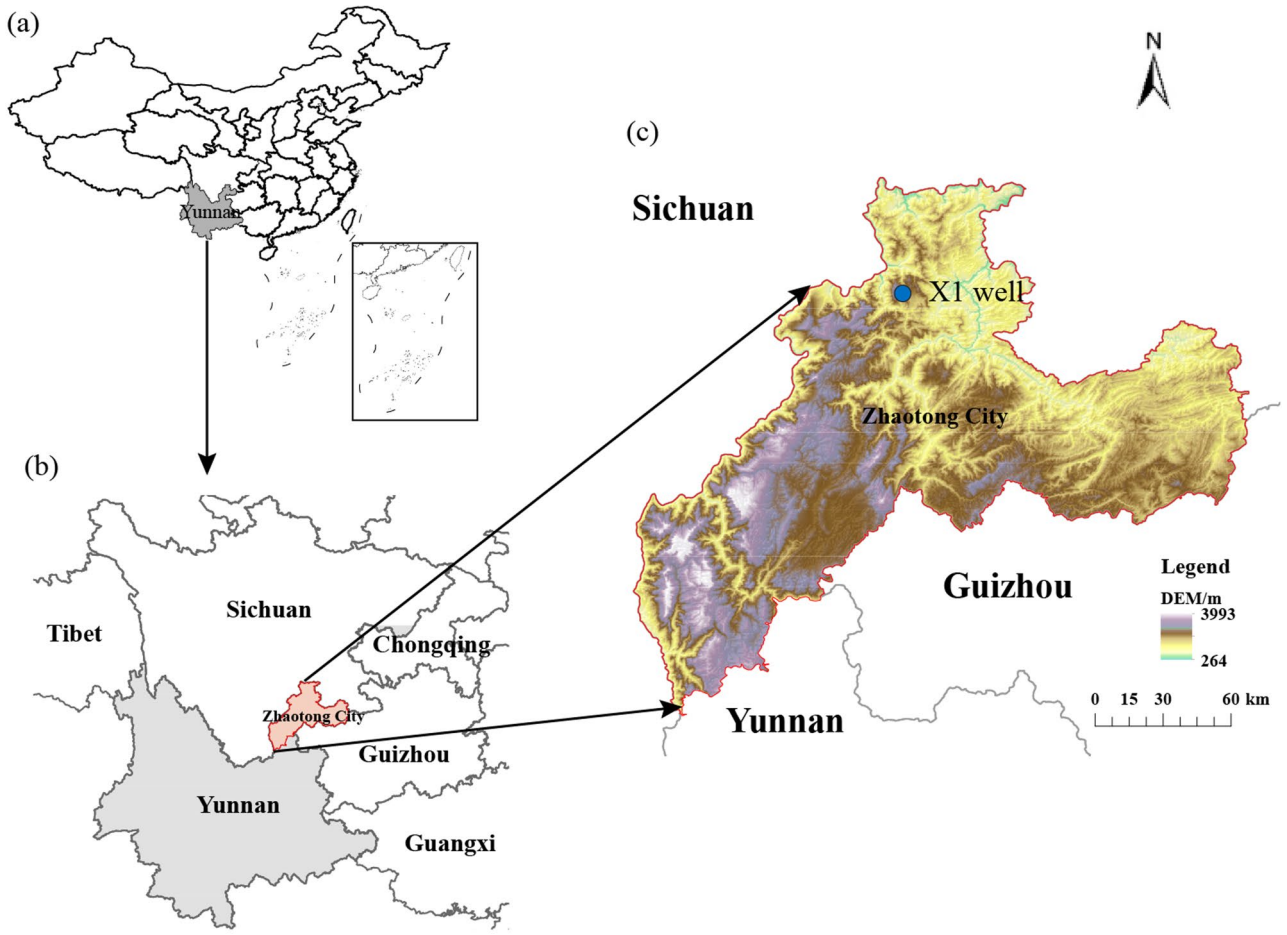
Location and research well of the Zhaotong shale gas demonstration area.

Shale gas has emerged as a significant source of energy in recent years, revolutionizing the global energy landscape^1^. The success of the North American “shale gas revolution” has profoundly transformed the global natural gas supply landscape. Shale gas is one of the key contributors to increasing China’s future energy reserves and production. The exploration and development of shale gas play a crucial role in alleviating China’s energy shortage. In recent years, China’s shale gas exploration has gradually shifted toward deeper and ultra-deep layers^2^. Significant advancements have been achieved in the Sichuan Basin and its periphery (Fig. 1). The demonstration blocks of Changning, Weiyuan, and Zhaotong gas shale play have cumulatively reported proven reserves of 10,610 × 10^8^ m^3^^3^.

The three-dimensional well factory combined with multi-stage, multi-cluster fracturing technology for long horizontal wells have overcome the limitations of low porosity and permeability in shale reservoirs, enabling the economic and efficient exploitation of shale gas^4^. The flowback fluid volume not only affects the overall efficiency of shale gas development but also contributes greatly to managing the environmental impact associated with wastewater treatment and disposal^5^. Many researchers have recognized the benefits of shutting in the well for a certain period of time to enhance the initial productivity of shale gas wells. In addition to the duration of the shut-in period, the control of flowback rate and fluid disposal methods are also crucial factors influencing the flowback volume and gas production of shale gas wells. Accurate prediction of the flowback fluid volume is crucial for optimizing the hydraulic fracturing process and ensuring its economic viability and environmental sustainability. Traditional methods for estimating the flowback fluid volume rely on empirical correlations and simplified mathematical models, which may lack accuracy and fail to capture the complex nonlinear relationships inherent in the hydraulic fracturing process^6^. Benson et al. developed a semi-analytical approach to analyze the flowback data of two-phase water and gas, particularly when spontaneous imbibition takes place^7^. It was discovered that during the flowback process, shale microfractures exhibit two-phase flow (gas and water), which renders the flow model used for conventional tight gas reservoirs inapplicable to the flowback behavior observed in shale gas reservoirs when compared with field data^7^. Xin et al. employed a specially designed device to estimate fracturing retention and flowback on overall development performance when large-scale fracturing operations in shale oil wells^8^. Experimental findings indicate a consistent decline in the flowback rate of fracturing fluid as the injected fluid volume increases^8^. This observation can be attributed to the improved retention of the injected fluid within the formation, resulting in a reduced flowback rate^8^. Zhang et al. proposed a methodology that combines flowback data and long-term production data to assess hydraulic fracture closure and variations in fracture characteristics^9^. Zhou et al. utilized a data-driven to investigate the association between gas production and flowback water in shale gas wells across both the wet and dry gas regions of northwestern West Virginia^10^. The study findings revealed contrasting relationships between produced gas and flowback water, with a positive correlation in the wet-gas region and a negative one in the dry-gas region^10^. Based on a comprehensive analysis of shale gas drainage and production test data from North America, Xie et al. discovered that reservoirs with a higher degree of natural fracture development and minimal disparity in situ stress tend to exhibit intricate fracture network systems created by artificial fracturing^11^. Consequently, these factors contribute to enhanced gas production and reduced flowback rates. Analyzing the data from the Changning block in southern Sichuan basin, it was identified that gas breakthrough time, gas breakthrough flowback rate, 30-day flowback rate, and maximum production flowback rate are significantly negatively correlated with test production^11^. A lower value of flowback characteristic parameters indicates a more favorable production outcome^11^. Lin et al. employed a BP neural network to establish correlations between petrophysical properties, flowback ratio, first-month cumulative production, and stimulated reservoir volume in shale gas^12^. Yet, these findings did not find better correlation relationships between SRV and first-month cumulative production.

The prediction of production in horizontal wells after hydraulic fracturing is a prerequisite for the precise design of fracturing parameters. Intelligent production prediction combines big data and artificial intelligence to dynamically forecast and analyze shale gas production, offering the potential to overcome the limitations of low efficiency in numerical simulation calculations and significantly improve prediction speed. Lee et al. conducted an analysis of time series databases related to shale gas production and identified a significant feature that influences production history^13^. These findings revealed that the production model incorporating both production history and the shut-in period as input features exhibited superior prediction accuracy compared to models utilizing a single feature (production history) or the traditional decline curve analysis (DCA) method^13^. Li et al. introduced an integrated model that combines the Bi-GRU and Sparrow Search Algorithm^14^. The experimental results demonstrated that the hybrid model outperformed traditional methods such as the DCA and conventional time series analysis methods in terms of prediction accuracy and robustness^14^. Yi et al. introduced a novel algorithm called weighted warping K-means clustering that consider the varying influences of different variables^15^. However, their study primarily focused on the impact of production pressure changes and did not fully explore the development of distinct time-series production prediction models across various scenarios.

Fracture-fluid flowback serves as a crucial indicator of shale gas well performance, but the impact of flowback on gas production and the subsequent influence of produced water on gas production under specific conditions remain uncertain. Leonard et al. examined six wells across three areas of the Barnett shale, revealing a negative correlation between higher fracture-fluid flowback and gas production^16^. Curry et al. conducted a comparison of two wells in the Marcellus gas shale play with comparable completion design and hydraulic fracturing^17^. These findings demonstrated that higher fracture-fluid flowback was associated with increased early production. Through the utilization of data mine and a distribution-free model, Zhou et al. aims to identify and illustrate the correlations between gas production and hydraulic fracture fluid flowback in the Marcellus across various spatial and temporal scales^10^. These findings demonstrate a positive correlation between gas production and fracture-fluid flowback in the wet gas region, whereas a negative correlation is observed in the dry gas region. However, due to insufficient field data from earlier research, the relationship between fracturing flowback and gas production in short and long-term could not be conclusively established. Both experimental and simulation studies have yet to offer a detailed insight into the fracture-fluid flow mechanism and production in gas shale.

A key bottleneck in characterizing such relationship between fracturing fluid and production stems from the difficulty of predicted approaches structures due to general methodological challenges and aspects relevant in the shale gas domain such as high dimensionality, complex underlying events, the presence of hidden/unmeasured variables, limited data and noise levels. Although existing data-driven methods have made progress in shale gas production forecasting, they often overlook the complex nonlinear coupling between fracturing fluid flowback and production and exhibit insufficient accuracy in handling irregular spatio-temporal data. These limitations restrict the precision of fracturing parameter optimization and increase uncertainty in environmental risk assessments. In this work we achieve accurate prediction of fluid flowback, and production based on the shale gas wells data in Zhaotong gas shale play in Sichuan basin by using CNN-Transformer model. This model incorporates multi-head attention and positional encoding mechanisms. We train and simulate the model using a dataset obtained from real shale gas production wells. The CNN-Transformer is a hybrid of CNN and Transformer models that capture the spatiotemporal dependence of fracturing flowback and production data and improve predictive robustness without sacrificing accuracy and efficiency. This work proposes a novel CNN-Transformer hybrid framework, which innovatively integrates the local sequence correlation capture of CNN with the global temporal attention mechanism of Transformers, specifically designed for joint prediction of hydraulic fracturing fluid flowback and shale gas production. The key innovations lie in^1^: dynamically handling the nonlinear path dependence of gas-water two-phase flow by extracting state information from historical fracturing flowback and shale gas production through attention modules^2^; introducing data fusion to enhance robustness against noise interference and environmental disturbances, surpassing the temporal limitations of traditional RNN-based models. The main contributions of this work include^1^: developing and validating the CNN-Transformer system, achieving state-of-the-art performance in flowback volume prediction outperforming CNN-LSTM and CNN-GRU-AM)^2^; significantly reducing analysis errors in production prediction, revealing the negative correlation mechanism between flowback rate and long-term production^3^; providing AI-driven decision tools for balancing economic efficiency and environmental sustainability in shale gas development, promoting the application of large models in petroleum engineering.

The structure of this paper is outlined: Sect. 2 preprocess the historical production and fracturing fluid flowback datasets. Section 3 explicitly describes the CNN, Transformer, and CNN-Transformer models. The predicted results, the analyses and comparison of CNN-LSTM, CNN-GRU-AM to verify the CNN-Transformer are reported in Sect. 4. This essay has a conclusion in Sect. 5.

## Statistical preprocessing

### Database generation

**Fig. 2:**
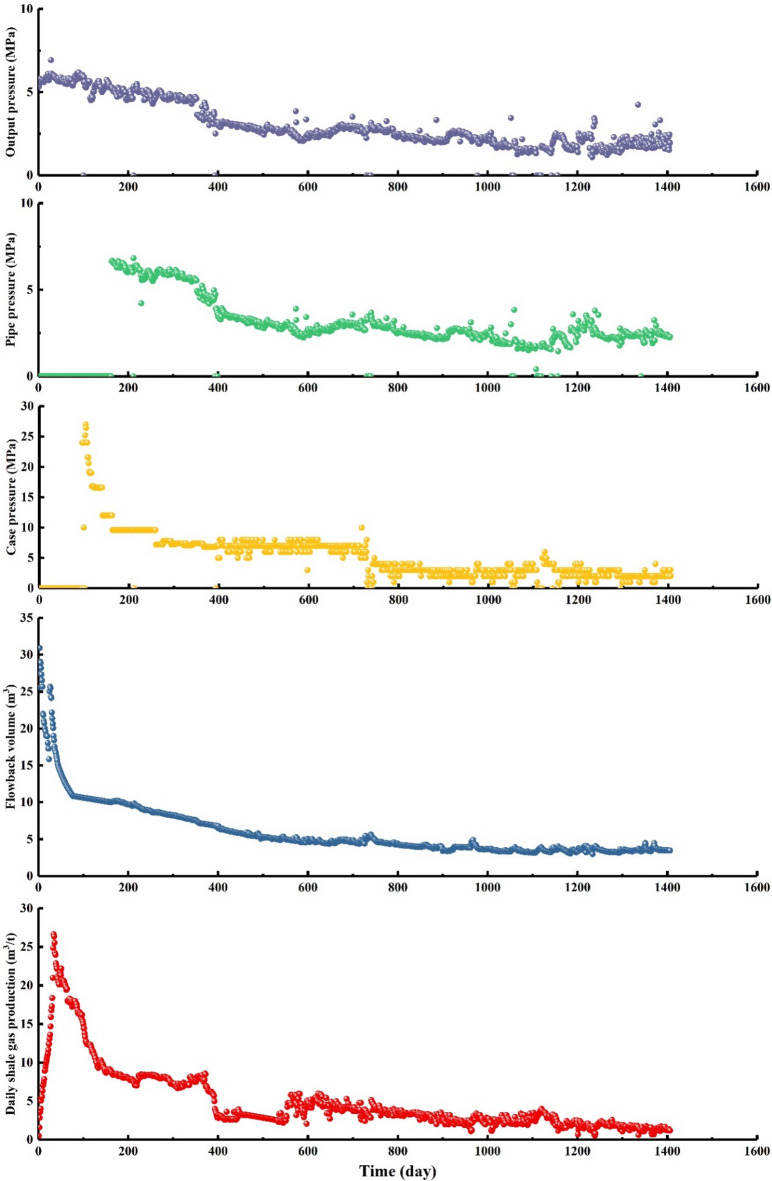
Raw data of X1 shale gas well include production, flowback volume, casing pressure, pipe pressure, output pressure.

The production, fracturing fluid flowback rate, casing pressure, pipeline pressure, and tubing pressure data were collected from X1 shale gas well in the Zhaotong shale gas demonstration area (Fig. 1). The dataset time ranges from 1 to 1406 days (Fig. 2). Given the diverse nature and uncertain characteristics of the aforementioned data types, various measures and methodologies have been employed to address these challenges. Engineering data encompasses various practical information from different subjects to minimize uncertainty, including casing pressure, pipeline pressure, tubing pressure. Due to the fact that the changes in the casing pressure, pipeline pressure, and output pressure eventually converge to the same constant (Fig. 2), conducting time series forecasting holds little significance. Therefore, these three variables will no longer be considered in this paper.

Production and flowback data, collected and monitored by sensors and calculators in the shale gas wells, undergo thorough verification, evaluation, and adjustment by field engineers and experts from petroleum research institutes. Despite efforts to minimize uncertainty and enhance data quality, some errors may still persist, such as systematic methodological errors. The experimental environment for this study was a computer running on a 64-bit Win10 operating system. The computer was equipped with an Intel Core i7-8300 H CPU, an NVIDIA GeForce GTX 4090 GPU, and 16GB of RAM. The software environment included Python 3.7.6 and the TensorFlow 2.3.1 deep learning framework.

### Preprocessing dataset

We incorporate comprehensive site-specific production and engineering factors into the deep-learning algorithms, which involve two important steps: preprocessing parameters and optimizing computing algorithms^4^. First, input parameters undergo data normalization to rescale them to a normal distribution within the range of 0–1. This normalization process enhances the training efficiency of computing algorithms. Typically, data preprocessing involves utilizing the following standardization expressions^4^:

1 $$y_{i} = \frac{{x_{i} - \mu \:}}{{\sigma \:}}$$

2 $$\:\mu\:=\frac{{\sum\:}_{i=1}^{n}\left({x}_{i}\right)}{n}\:\:$$

3 $$\:\sigma\:=\sqrt{\frac{{\sum\:}_{i=1}^{n}{({x}_{i}-\mu\:)}^{2}}{n}}$$

where the normalized value (*y*_*i*_) of each parameter (*x*_*i*_) is calculated using the mean value (*u*) and standard deviation (*σ*) of the parameters, *n* represents the total number of parameters. This normalization process ensures that the parameters are rescaled to a standard range of 0–1. Subsequently, the computing algorithms are optimized to enhance their performance.

## Method

We evaluated three representative types of deep learning algorithms, CNN-LSTM, CNN-GRU-AM, and CNN-Transformer. These methods encompass a range of classic approaches in deep learning. CNNs are a class of deep learning models designed to process data with a grid-like topology, such as images (Figure A1). They automatically learn and extract hierarchical features, like edges and textures, through a series of convolutional layers. LSTM networks are a type of RNN specifically engineered to handle sequential data (Figure A2). By employing a unique gating mechanism, LSTMs can selectively remember or forget information over long periods, thereby addressing the vanishing gradient problem (Figure A3). GRUs is a simplified and computationally more efficient version of LSTMs (Figure A4). They combine the forget and input gates into a single update gate and merge the cell state and hidden state, which allows them to capture long-term dependencies with fewer parameters (Figure A4). The CNN-LSTM model combines the strengths of both architectures (Figure A5). The CNN component first extracts relevant spatial features from the input data, which are then passed to the LSTM component. The LSTM subsequently models the temporal relationships within these extracted features. The CNN-GRU-AM model represents a more complex hybrid architecture that integrates a CNN, a GRU, and an AM (Figure A6). A detailed description of CNN, LSTM, CNN-LSTM, and CNN-GRU-AM is provided, and they are placed in the appendix of this paper.

### Overall framework

Time series data’s local correlation manifests through continuous changes within a time interval, effectively captured by convolutional filters. Hence, researchers have delved into employing CNNs for time series modeling and forecasting. Notably, dilated causal convolution stands as the predominant method utilized in this context. Our framework consists of an CNN model that two one-dimensional convolution layers extract underlying features, as well as a Transformer model that mine and preserve the temporal of the time series data and transfer the CNN output to the encoder layers^18^. The encoding results are fed into a decoder layers, followed by the target sequence, which is stacked with shallow high-dimensional features.

Shale gas production and fracturing fluid flowback data can change significantly over time due to various events, including scheduled equipment maintenance, well shutdown, well stoppage, and extreme weather conditions. Consequently, both outliers and trend change points are closely connected to the surrounding data structure. This approach uses CNNs for extracting features from shale gas production and fracturing flowback sequences and employs convolution kernels of varying sizes to capture more detailed local information.

**Fig. 3:**
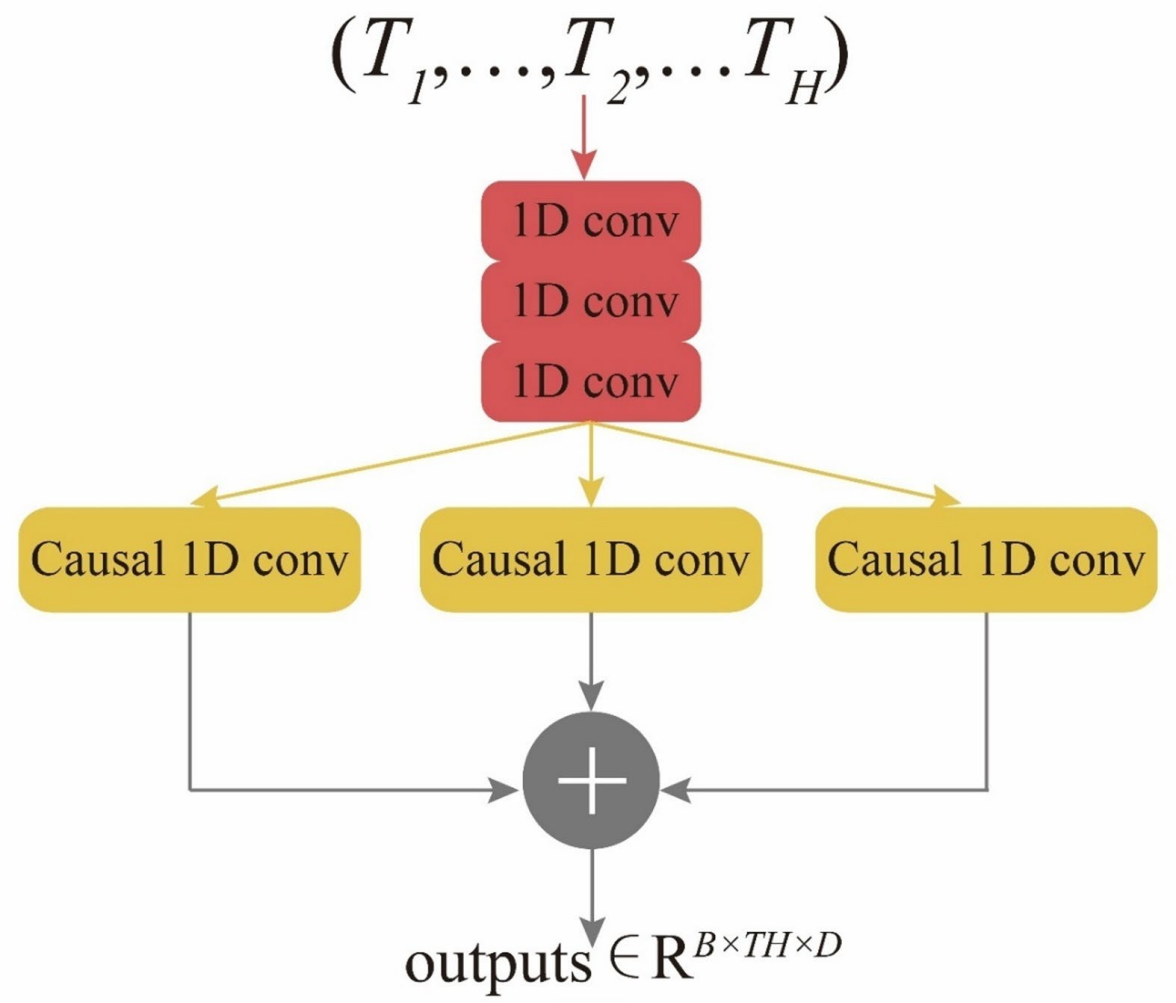
The basic structure of sequence embedding with 1D convolution network and canonical convolutions.

To prevent future information disclosure, we use 1D causal convolution for sequence embedding. We use causal convolutions to construct a pure convolutional network as sequence embedding layer, so that the improved Transformer can be applied to the task of shale gas production and fracturing flowback (Fig. 3). Figure 3 depicts the proposed sequence embedding layer. The stacked 1D causal convolutions, using a kernel size of 1, can scale sequence features while preserving the original sequence length. To improve the sequence’s locality, 4 parallel 1D causal convolutions are applied. The features extracted through these convolutions provide rich representations^19^. The input is shale gas production or fracturing fluid flowback data can be defined as:

4 $$\:V\in\:\:{R}^{B\times\:{T}_{H}\times\:1}\:\:\:\:\:\:\:\:\:\:\:\:\:\:\:\:\:\:\:\:\:\:\:\:\:\:\:\:\:\:\:\:\:\:\:\:\:\:\:\:\:\:\:\:\:\:\:\:\:\:\:\:\:\:\:\:\:\:\:\:\:$$

where *V* is the input tensor for shale gas production and fracturing flowback data; *B* represents the batch size; *T*_*H*_ represents the length of historical time steps; 1 indicates the feature dimension for each time step, which is one-dimensional shale gas production and fracturing flowback data, implying that each time step has only one feature value.

**Fig. 4:**
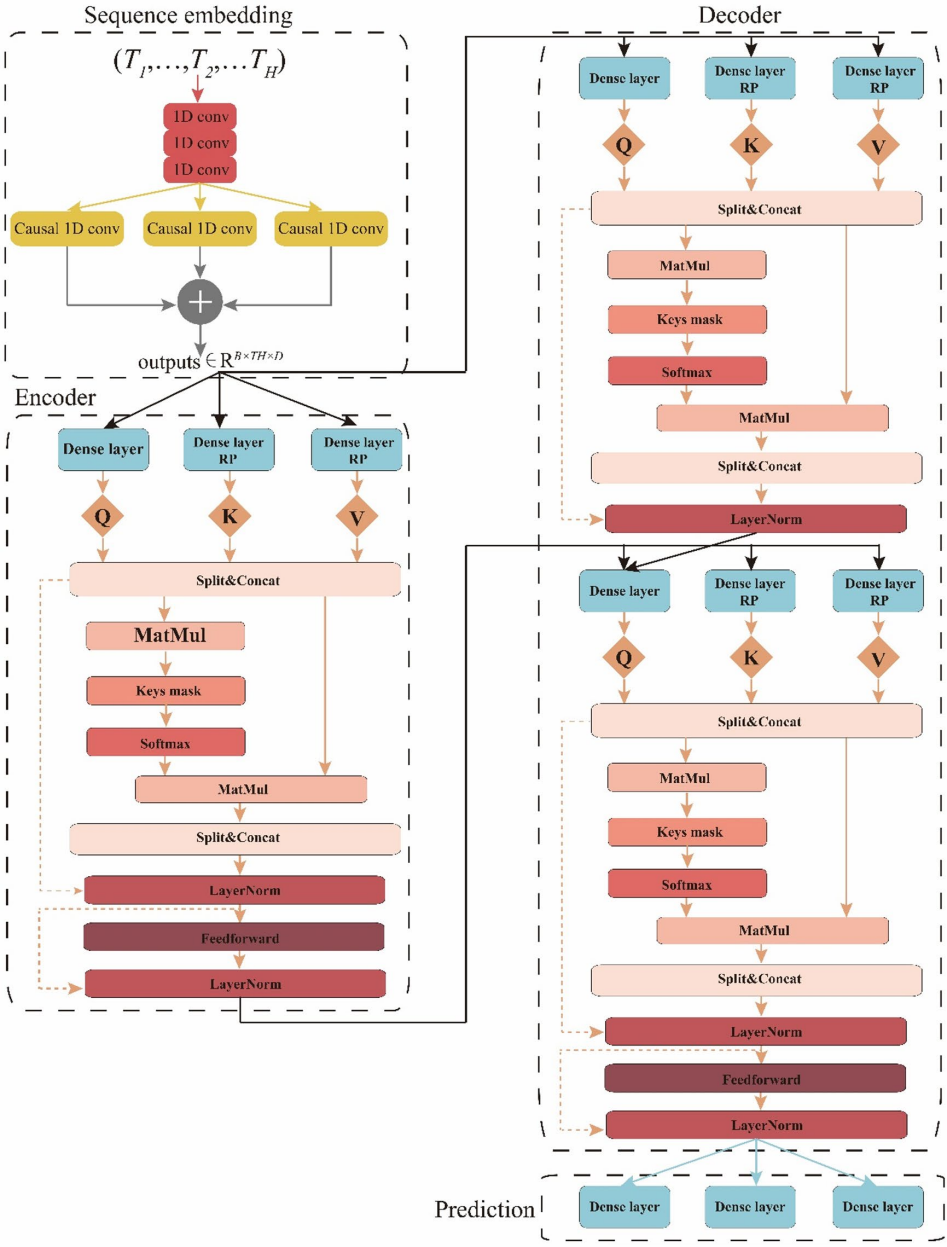
The basic framework of CNN-Transformer.

The advancements involve using a pure convolutional network to construct a sequence embedding layer (Fig. 4), which captures the local correlation between shale gas production and fracturing flowback data. The proposed model employs an encoder-decoder structure. The encoder and decoder each incorporate multiple identical sub-layers, with the additional need to address overfitting and memory overflow. To prevent information loss during learning, skip connections are integrated within both the encoder and decoder blocks (Fig. 4). To handle variable-length input sequences and prevent future information leakage, we utilize a key mask and a causal mask, respectively (Fig. 4). The CNN-Transformer framework can be described as follows:

The input to the CNN-Transformer consists of the historical shale gas production and fracturing flowback data *H*$\:\in\:{R}^{1\times\:B\times\:{T}_{H}}$ and a zeros matrix *P*$\:\in\:{R}^{1\times\:B\times\:{T}_{F}}$^20^.

where *H* represents a matrix (or tensor) indicating the historical shale gas production and fracturing flowback data. *P* is a zeros matrix with the same shape as the output tensors. *T*_*F*_ represents the number of future time steps; The shape of this matrix is *B*×*T*_*F*_×1, indicating that there are *B* samples, each with *T*_*F*_ time steps, and each time step has 1 feature. Future information cannot be used as input in the inference phase. Therefore, a slight modification is needed for *P*^18^.

5 $$P \leftarrow \left[ {H\left[ {:, - 1:,:} \right] \circ P\left[ {:,: - 1,:} \right]} \right]$$

where *H* [:,−1:,:] represents extracting the data from the last time step in *H*; *P*[:,:−1,:] represents removing the data from the last time step in *P*; ∘ represents the concatenation operation.

Through this operation, the new *P* will be formed by concatenating the last time step of *H* with the first *T*_*F*_−1-time steps of *P*. This adjustment ensures that future information is not used during the inference phase.

Sequence embedding projects *H* and *P* into a high-dimensional space. It can be defined as^18^:

6 $${H^{(0)}} \in {R^{B \times {T_H} \times D}}$$

7 $${P^{(0)}} \in {R^{B \times {T_F} \times D}}$$

where *D* denotes the number of hidden units.

The projected feature *H*^(0)^ is input into the encoder layer, while *P*^*(0)*^ is fed into the decoder layer. *H*^*(1)*^ and *P*^*(0)*^ are then fed into the decoder to get the result *P*^(1)^.

The decoder forecasts shale gas production and fracturing flowback using an autoregressive approach^18^.

8 $${P^{(1)}} \in {R^{B \times {T_F} \times D}}$$

The tensor *P*^*(1)*^ is utilized to update *P*^*(1)*^ along the temporal dimension. The above steps need to be repeated *T*_*F*_​ times. This means the model will predict and update *P*^*(1)*^ at each time step.

### Encoder

The encoder layer processes the feature of historical shale gas production and fracturing flowback tensor following its projection by the sequence embedding layer (Fig. 4). The encoder layer is composed of multi-head attention (MMA), feed forward network (FFN), and layer normalization layer (LN). In MMA, we incorporate an offset in the linear projection to account for the relative positions at various time points. FFN applies a position-wise transformation to achieve changes in the tensor dimension. LN processes the tensor using batch normalization to speed up convergence.

The MMA calculates feature tensors through a modified scaled Dot-Product attention. Multi-heads involve splitting queries, keys, and values ∈ ${R^{B \times {T_H} \times D}}$into *N* sub-tensor. The outputs from each head are concatenated to form the result of MMA. We introduced the key mask method in MMA, allowing it to handle input sequences of any length or incomplete sequences.

The specifics of key mask for encoder (KME) are outlined as follows^18^:

The input include the *Att* and *enc. Att* represents the multi-head attention score matrix prior to softmax normalization, *enc* is the input after processing through the sequence embedding block^18^. The *Att* can be defined as:

9 $$Att \in R^{{(B \times N) \times T_{H} \times T_{H} }}$$

where *Att* is the multi-head attention score matrix; *N* is the number of attention heads computed in parallel.

The specific formula for *enc* is as follows^18^:

10 $$enc \in R^{{B \times T_{H} \times D}}$$

The mask memory can be described by^18^:

 .

11 $$mask memory \in R^{{B \times T_{H} \times 1}}$$

The key mask is generated by summing the absolute values of all feature dimensions and then expanding its shape to match the time dimension of the input. The key mask can be described by^18^:

12 $$key{\text{ }}mask{\text{ }} \leftarrow {\text{ }}\exp and\_\dim s{\text{ }}(sign\:\sum {\:_{{d = 1}}^{D} } |enc[:,\::,\:d]|),{\text{ }}axis = - 1)^{{~~~~~~~~~~~~~~~~~~~~}}$$

Transpose mask is transposed to match the dimensional requirements of subsequent operations. The transpose mask is defined by^20^:

13 $$key{\text{ }}mask{\text{ }} \leftarrow {\text{ }}transpose(key{\text{ }}mask,{\text{ }}[0,{\text{ }}2,{\text{ }}1])$$

Expanding the mask to match the dimensions of the attention score matrix. It can be defined as^20^:

14 $$key{\text{ }}mask{\text{ }} \leftarrow {\text{ }}tile(key{\text{ }}mask,{\text{ }}[N,{\text{ }}TH,{\text{ }}1])$$

Here, we assume the initial dimensions of the key mask are (*B*,*1*, *T*_*H*_). The tile function is used to repeat an array along specified dimensions. Its syntax is tile (input, reps), where input is the input array, and reps are the number of repetitions for each dimension. The tile function’s purpose is to copy the key mask along specific dimensions, thereby extending its shape. After this step, the dimensions of the key mask will become (*B×N*, *T*_*H*_, *T*_*H*_), matching the dimensions of the multi-head attention score matrix *Att* (*B×N*, *T*_*H*_, *T*_*H*_). By using the tile function to expand the key mask to the same dimensions as the attention score matrix, each attention head will have a corresponding mask, ensuring that the mask is correctly applied in subsequent calculations.

Create a padding matrix with a value of − 2^32^+1 to fill invalid positions in the mask^20^.

15 $$padding{\text{ }} \leftarrow {\text{ }}ones{\text{ }}\left( {\left( {B{\text{ }} \times N,T_{H} ,T_{H} } \right)} \right) \times ( - {\text{ }}2^{{32}} + {\text{ }}1)$$

We create a matrix filled with ones using the one’s function. The parameter of the ones function is the shape of the matrix (*B×N*, *T*_*H*_, *T*_*H*_). Therefore, ones((*B*×*N*, *T*_*H*_, *T*_*H*_)) creates a tensor with shape (*B*×*N*, *T*_*H*_, *T*_*H*_), where all elements are 1. Padding value − 2^32^+1 is a very large negative number, which is typically used to indicate invalid or irrelevant positions in calculations, as such a large negative number will be close to zero in attention calculations and hence be ignored. Multiplying the matrix of ones by this large negative number results in a padding matrix of shape (*B×N*, *T*_*H*_​, *T*_*H*_​), where all elements have the value − 2^32^+1.

Replace positions with 0 in the key mask with the padding value^20^.

16 $$key{\text{ }}mask{\text{ }}[key{\text{ }}mask{\text{ }} = {\text{ }} = {\text{ }}0]{\text{ }} \leftarrow {\text{ }}padding$$

Assume key mask is a tensor with the shape (*B×N*, *T*_*H*_, *T*_*H*_). It is used in the attention mechanism to mask out invalid positions. Equation 16 means replacing all positions in key masks that are 0 with the padding value.

The specific steps are as follows: The expression key mask = = 0 generates a Boolean matrix of the same shape as key mask, where positions with the value 0 correspond to true, and other positions correspond to false. By using key mask [key mask = = 0] = padding, we replace the key mask elements at positions where the boolean matrix is true with the padding value. By performing the step key mask [key mask = = 0] = padding, we replace all positions in key mask that are 0 with the padding value − 4,294,967,295.

Replace positions in the key mask that are not 0 with the corresponding attention scores^20^.

17 $$key{\text{ }}mask{\text{ }}[key{\text{ }}mask{\text{ }} \ne {\text{ }}0]{\text{ }} \leftarrow Att$$

Assume key mask is a tensor with the shape (*B*×*N*, *T*_*H*_, *T*_*H*_). It is used in the attention mechanism to mask out invalid positions. Assume *Att* is a tensor with the shape (*B×N*, *T*_*H*_, *T*_*H*_) that stores the attention scores. Equation 17 means replacing all positions in key mask that are not 0 with the corresponding values from *Att*. The expression key mask ≠ 0 generates a Boolean matrix of the same shape as key mask, where positions with a value not equal to 0 correspond to true, and other positions correspond to false.

By using key mask [key mask ≠ 0] = *Att* [key mask ≠ 0], we replace the key mask elements at positions where the boolean matrix is True with the corresponding values from *Att*. By performing the step key mask [key mask ≠ 0] = *Att* [key mask ≠ 0], we replace all positions in key mask that are not 0 with the corresponding attention scores from *Att*. This ensures that in subsequent calculations, only the valid positions will affect the final attention results.

Finally, assign the processed key mask to the attention score matrix^20^.

18 $$Att{\text{ }} \leftarrow key{\text{ }}mask$$

By performing the step *Att* ← key mask, we assign the processed key mask to *Att*, so that *Att* contains the updated attention score matrix.

The FFN consists of two dense layers. The first layer utilizes the LeakyReLU activation function, while the second layer performs a linear mapping. It can be defined as^21^:

19 $$FFN{\text{ }}(x){\text{ }} = {\text{ }}Leaky\text{Re} lu{\text{ }}(xW1{\text{ }} + {\text{ }}b1)W2{\text{ }} + {\text{ }}b2$$

where *x*${R^{B \times {T_H} \times D}}$is the input of FFN. *W*_*1*_$\:\in\:$*R*^*D×4D*^, *b*_*1*_$\:\in\:$*R*^*4D*^, *W*_*2*_$\:\in\:$*R*^*D×4D*^, *b*_*2*_$\:\in\:$*R*^*D*^ are trainable parameters. These denote the dimensions of the weight matrices and bias vectors. The first layer expands the dimensionality from *D* to 4*D*, and the second layer reduces it back from 4*D* to *D*. *W*_*1*_​ is the weight matrix for the first dense layer; *b*_*1*_​ is the bias vector for the first dense layer. LeakyReLU is the activation function applied after the first dense layer. *W*_*2*_​ is the weight matrix for the second dense layer. *b*_*2*_​ is the bias vector for the second dense layer. Skip connection is a reference to the ResNet architecture, which involves adding the input *x* to the output of a layer to help with gradient flow and prevent the vanishing gradient problem. Skip connections and LN are utilized to enhance the learning capabilities of deep networks.

The details of the encoder layer are outlined as follows^21^:

20 $$enc{\text{ }}memory \in R^{{B \times D \times T_{H} }}$$

where enc memory is encoded memory tensor.

21 $$mask{\text{ }}memory \in R^{{B \times 1 \times T_{H} }}$$

where mask memory is masked memory tensor.

The query (*Q*), key (*K*), and value (*V*) matrices can be defined as^21^:

22 $$Q \leftarrow enc\underline{{}} \times Wq,WQ \in R^{{D \times D}}$$

23 $$K \leftarrow enc\underline{{}} \times W_{k} + b_{k} ,W_{k} \in \:R^{{D \times D}} ,b_{K} \in \:R^{D}$$

24 $$V \leftarrow enc\underline{{}} \times W_{v} + b_{v} ,W_{v} \in \:R^{{D \times D}} ,b_{v} \in \:R^{D}$$

*Q* is obtained by applying a linear transformation to enc with a weight matrix *W*_*q*​_. *K* is obtained by applying a linear transformation to enc and adding a bias term *b*_*k*_​. *V* is obtained by applying a linear transformation to enc and adding a bias term *b*_*v*​_.

To support the multi-head attention mechanism, the *Q*, *K*, and *V* matrices are concatenated and split as follows^21^:

25 $$Q{\text{ }} \leftarrow concat{\text{ }}(split{\text{ }}(Q,{\text{ }}N,{\text{ }}axis{\text{ }} = {\text{ }} - 1),{\text{ }}axis{\text{ }} = {\text{ }}0)$$

26 $$K{\text{ }} \leftarrow concat{\text{ }}(split{\text{ }}(K,{\text{ }}N,{\text{ }}axis{\text{ }} = {\text{ }} - 1),{\text{ }}axis{\text{ }} = {\text{ }}0)$$

27 $$V{\text{ }} \leftarrow concat{\text{ }}(split{\text{ }}(V,{\text{ }}N,{\text{ }}axis{\text{ }} = {\text{ }} - 1),{\text{ }}axis{\text{ }} = {\text{ }}0)$$

The split (*Q*, *N*, axis = −1) splits *Q* along the last dimension into *N* parts. Concat (split (*Q*, *N*, axis = −1), axis = 0) concatenates the split *Q* matrices along a new dimension. Similarly, *K* and *V* undergo the same operations.

The attention score matrix *Att* using the scaled dot-product attention formula can be defined as^21^:

28 $$\:Att \leftarrow \frac{{Q\_ \times \:(K\_)^{T} }}{{\sqrt D }}$$

Use the KME function to apply masking to *Att* and enc, resulting in *Att* and mask memory.

29 $$Att,{\text{ }}mask{\text{ }}memory{\text{ }} \leftarrow KME{\text{ }}(Att,{\text{ }}enc)$$

Apply the Softmax operation to the attention score matrix *Att* to compute the attention weights^22^.

30 $$outputs{\text{ }} \leftarrow Soft\max {\text{ }}(Att)$$

Multiply the attention weights *Att* by the value matrix *V_*. This operation is equivalent to performing a weighted sum of the values, resulting in the weighted output^22^.

31 $$outputs{\text{ }} \leftarrow outputs{\text{ }} \times V\underline{{}}$$

Split (outputs, *N*, axis = 0) splits outputs into *N* parts along the 0th dimension. Concat(split(outputs, *N*, axis = 0), axis = −1) concatenates the split outputs along the last dimension.

32 $$outputs{\text{ }} \leftarrow concat{\text{ }}(split{\text{ }}(V,{\text{ }}N,{\text{ }}axis{\text{ }} = {\text{ }} - 1),{\text{ }}axis{\text{ }} = {\text{ }}0)$$

Apply residual connection and layer normalization to the concatenated outputs. outputs + enc is the residual connection, adding the input enc to the outputs.

33 $$outputs{\text{ }} \leftarrow LN{\text{ }}(outputs{\text{ }} + {\text{ }}enc)$$

FFN (outputs) is the output of the feed-forward network. Perform a residual connection by adding the original outputs to the output of the FFN, followed by layer normalization.

34 $$outputs{\text{ }} \leftarrow {\text{ }}LN{\text{ }}(FFN{\text{ }}(outputs){\text{ }} + {\text{ }}outputs)$$

Update the encoder input enc to the current layer’s output outputs.

35 $$enc{\text{ }} \leftarrow outputs$$

### Decoder

The decoder layer includes multi-head self-attention (MSA) and multi-head interactive attention (MIA) (Fig. 4). MSA handles the calculation of attention distribution across time nodes in the future sequence. MIA is responsible for calculating the interactive attention distribution of historical sequence and future sequence. The attention mechanism distribution still conforms to Eq. 39∽Eq. 40^22^.

36 $$\:{q}_{i}={x}_{i}{W}_{q}$$

37 $$\:{k}_{j}={x}_{j}{W}_{k}+{b}_{ij}^{k}$$

38 $$\:{v}_{j}={x}_{j}{W}_{v}+{b}_{ij}^{v}$$

39 $$\:{a}_{ij}=softmax\:\left(\frac{{q}_{i}{k}_{j}^{T}}{\sqrt{{D}_{k}}}\right)$$

40 $$\:{O}_{i}=\sum\:_{j}{a}_{ij}{v}_{j}$$

where $\:{b}_{ij}^{k}$is the bias of keys; *x*_*i*_ *and x*_*j*_ serve as the input of the attention layer with the shape *R*^*D*^; *q*_*i*_, *k*_*j*_, *v*_*j*_ are quires, keys, and values; *W*_*q*_, *W*_*k*_, *W*_*v*_ is the weight matrix of quires, keys, and values. *D*_*k*_ is the dimension number of keys; *a*_*ij*_ is the attention score between *i* and *j*; *O*_*i*_ represents the outputs.

In the MSA, the computation process closely mirrors that of encoder layer. Additionally, a new causal mask integrated with the key mask handles the masking in MSA, preventing future information leakage and promoting more stable training. This means that the shale gas production and fracturing flowback at the next step depends solely on past and present states, highlighting a clear causal relationship. Consequently, we refer to this masking method in the decoder layer as the causal mask (CMD). Detailed implementation of CMD is presented in are outlined as follows:

The input is *Att* $\:\in\:$ *R*^*(B×N)×TF×TF*^.

Initialize the causal mask, the causal mask is a matrix with dimensions T_F_×T_F_^22^.

41 $$causalmask \leftarrow ones{\text{ }}(T_{F} ,{\text{ }}T_{F} )$$

Transform the causal mask into a lower triangular matrix, retaining only the elements on and below the main diagonal while setting all other elements to zero^22^.

42 $$causal{\text{ }}mask{\text{ }} \leftarrow Lower{\text{ }}Triangular{\text{ }}(causal{\text{ }}mask)$$

Use *expand_dims* to add a new dimension at the 0th axis of the causal mask, changing its shape to 1×T_F_×T_F_​. Then, use the tile function to repeat it *B*×*N* times along the 0th axis, resulting in a tensor with shape (*B*×*N*)×T_F_×T_F_​.

43 $$causal{\text{ }}mask{\text{ }} \leftarrow {\text{ }}tile{\text{ }}(\exp and\_\dim s{\text{ }}(causal{\text{ }}mask,{\text{ }}0),{\text{ }}[(B \times N),{\text{ }}1,{\text{ }}1])$$

Create a tensor of shape (B×*N*)×T_F_×T_F_ filled with the value − 2^32^+1 to be used as padding^22^.

44 $$padding{\text{ }} \leftarrow {\text{ }}ones{\text{ }}((B{\text{ }} \times N),{\text{ }}T_{F} ,{\text{ }}T_{F} ){\text{ }} \times \left( { - 2^{{32}} + 1} \right)$$

Replace all positions in the causal mask where the value is 0 with the padding value^22^.

45 $$causal{\text{ }}mask{\text{ }}[causal{\text{ }}mask{\text{ }} = {\text{ }} = {\text{ }}0]{\text{ }} \leftarrow {\text{ }}padding$$

Replace all non-zero values in the causal mask with the corresponding values from the input attention matrix (*Att*).

46 $$causal{\text{ }}mask{\text{ }}[causal{\text{ }}mask{\text{ }} \ne 0]{\text{ }} \leftarrow Att$$

Assign the updated causal mask to *Att* to obtain the updated attention matrix.

47 $$Att{\text{ }} \leftarrow causal{\text{ }}mask$$

Return the updated attention matrix (*Att*).

MIA mechanism processes each attention head in a loop, computing the query, key, and value matrices, followed by masking, to ultimately produce the weighted output. *M* is the number of heads in multi-head attention. *N* is a specific parameter. The details of MIA can be outlined as follows:

The input includes mask memory, enc memory, dec memory^22^.

48 $$mask{\text{ }}memory{\text{ }} \in R^{{B \times 1 \times TH}}$$

49 $$enc{\text{ }}memory{\text{ }} \in R^{{B \times TH \times D}}$$

50 $$dec{\text{ }}memory{\text{ }} \in R^{{B \times TF \times D}}$$

Query matrix (*Q*) is calculated by multiplying the dec memory by the query weight matrix (*W*_*q*_).

51 $$Q \leftarrow dec{\text{ }}memory \times W_{q} ,W_{q} \in R^{{D \times D}}$$

Key matrix (*K*) is calculated by multiplying the encoder memory by the *W*_*k*_ and added the *b*_*k*_.

52 $$K \leftarrow {\text{ }}enc{\text{ }}memory{\text{ }} \times W_{k} {\text{ }} + {\text{ }}b_{k} ,{\text{ }}W_{k} {\text{ }} \in R^{{D \times D}} ,b_{k} \in R^{D}$$

Value matrix (*V*) is calculated by multiplying the value weight matrix (*W*_*v*_) and added the corresponding biases (*b*_*v*_).

​ 

53 $$V \leftarrow {\text{ }}enc{\text{ }}memory{\text{ }} \times W_{v} {\text{ }} + {\text{ }}b_{v} ,{\text{ }}W_{v} {\text{ }} \in R^{{D \times D}} ,b_{v} \in R^{D}$$

Split *Q*, *K*, and *V* into *N* parts, then concatenate them along a new dimension^19^.

54 $$Q\_{\text{ }} \leftarrow {\text{ }}concat(split(Q,{\text{ }}N,{\text{ }}axis = - 1),{\text{ }}axis{\text{ }} = {\text{ }}0)$$

55 $$K\_{\text{ }} \leftarrow {\text{ }}concat(split(K,{\text{ }}N,{\text{ }}axis = - 1),{\text{ }}axis{\text{ }} = {\text{ }}0)$$

56 $$V\_{\text{ }} \leftarrow {\text{ }}concat(split(V,{\text{ }}N,{\text{ }}axis = - 1),{\text{ }}axis{\text{ }} = {\text{ }}0)$$

Use the scaled dot-product attention formula to compute attention weights.

 

57 $$Att \leftarrow \:\frac{{Q_{\_} \left( {K_{\_} } \right)^{T} }}{{\sqrt D }}$$

Equations 58–61 expand the mask and set invalid positions to a very small value, making them close to zero after Softmax^19^.

58 $$key{\text{ }}mask{\text{ }} \leftarrow {\text{ }}tile(mask{\text{ }}memory,{\text{ }}[N,{\text{ }}T_{F} ,{\text{ }}1])$$

59 $$padding{\text{ }} \leftarrow {\text{ }}ones(B_{N} ,{\text{ }}T_{F} ,{\text{ }}T_{H} ){\text{ }} \times ( - {\text{ }}2^{{32}} + {\text{ }}1)$$

60 $$key{\text{ }}mask[key{\text{ }}mask{\text{ }} = {\text{ }} = {\text{ }}0]{\text{ }} \leftarrow {\text{ }}padding$$

61 $$key{\text{ }}mask[key{\text{ }}mask{\text{ }} \ne 0]{\text{ }} \leftarrow {\text{ }}Att$$

62 $$Att{\text{ }} \leftarrow {\text{ }}key{\text{ }}mask$$

Apply Softmax to the attention weights to obtain the weight matrix.

63 $$outputs{\text{ }} \leftarrow {\text{ }}Soft\max (Att)$$

Perform a weighted sum of the value matrix using the attention weight matrix.

64 $$outputs{\text{ }} \leftarrow {\text{ }}LN(outputs{\text{ }} + {\text{ }}dec{\text{ }}memory)$$

65 $$outputs{\text{ }} \leftarrow {\text{ }}outputs \times V\underline{{}}$$

Apply layer normalization to the output and process it through a feedforward neural network^19^.

66 $$outputs{\text{ }} \leftarrow {\text{ }}LN(FFN(outputs){\text{ }} + {\text{ }}outputs)$$

Update the output into the decoder memory for use in the next iteration.

67 $$dec{\text{ }}memory{\text{ }} \leftarrow {\text{ }}outputs$$

## Results and discussions

To verify the algorithm and evaluate its accuracy, a selection of shale gas wells from Zhaotong shale gas area, comprising production and flowback volume, were utilized as the basis for testing the proposed approach. The division of training and prediction sets for each network test was based on random sampling, maintaining a ratio of approximately 80%:20%. This approach was implemented to ensure optimal training set effectiveness while mitigating the risks of both overfitting and underfitting.

### K-fold cross-validation

K-fold cross-validation is a commonly used method for evaluating and fine-tuning models. It reduces the randomness in data partitioning and improves the model’s generalization ability. In this study, a 10-fold cross-validation approach is employed, where the data is divided into 10 subsets, and the cross-validation process is repeated for 10 rounds. In each round, the data is randomly split into complementary subsets. The model is trained on the training set and validated on the test set, and the average performance metrics from the 10 rounds serve as an estimate of the model’s prediction accuracy.

Common regression evaluation metrics include Root Mean Squared Error (RMSE) and R^2^. RMSE represents the difference between the model’s predicted values and the actual values, reflecting the accuracy of the model’s predictions. Specifically, RMSE is the square root of the mean of the squared prediction errors. RMSE is more sensitive to larger errors, as squaring the errors amplifies the impact of bigger discrepancies. The smaller the RMSE, the lower the model’s prediction error and the higher its accuracy; conversely, a larger RMSE indicates greater prediction error in the model. *R*^*2*^ represents the extent to which the model explains the variation in the observed data. The value of *R²* ranges from 0 to 1, indicating the proportion of the dependent variable’s variation explained by the predictive model. *R*^*2*^ = 1indicates that the model perfectly fits the data, meaning all predicted values are exactly the same as the actual values, and the model explains all the variation. *R*^*2*^ = 0 means that the model cannot explain any variation in the data, and its predictions are no better than simply using the average value. 0 < *R*^*2*^ < 1 suggests that the model can explain part of the variation in the data, with values closer to 1 indicating stronger explanatory power. The formulas of RMSE and R^2^ are as follows^23^:

68 $$RMSE=\sqrt {\frac{1}{N}{{\sum\nolimits_{{i=1}}^{N} {({Y_i} - {Z_i})} }^2}}$$

69 $$R^{2} = 1 - \:\frac{{\sum {\:_{{i = 1}}^{n} } \left( {Y_{{i - }} Z_{i} } \right)^{2} }}{{\sum {\:_{{i = 1}}^{n} } (Y_{i} - \mathop {Y_{i} }\limits^{ - } )^{2} }}$$

### Fracturing fluid flowback prediction results

#### Fracturing fluid flowback predicted by CNN-LSTM

**Fig. 5:**
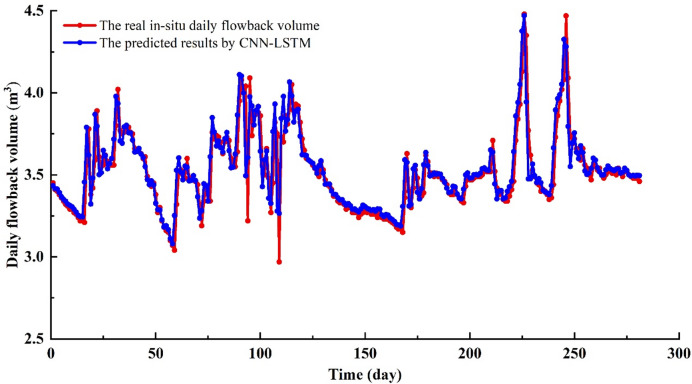
The fracturing fluid flowback predicted results by CNN-LSTM vs. in-situ data.

**Fig. 6:**
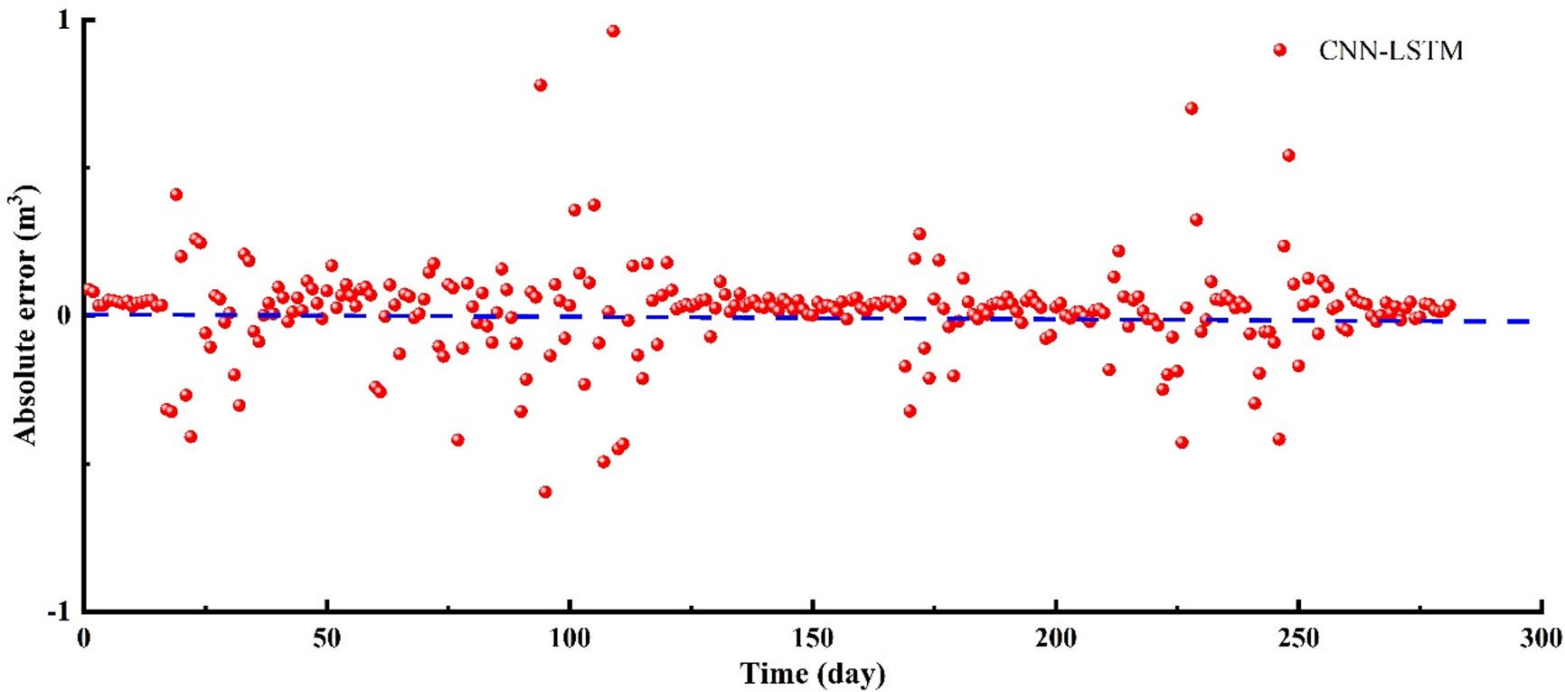
The absolute error of fracturing fluid flowback between CNN-LSTM and in-situ data.

**Fig. 7:**
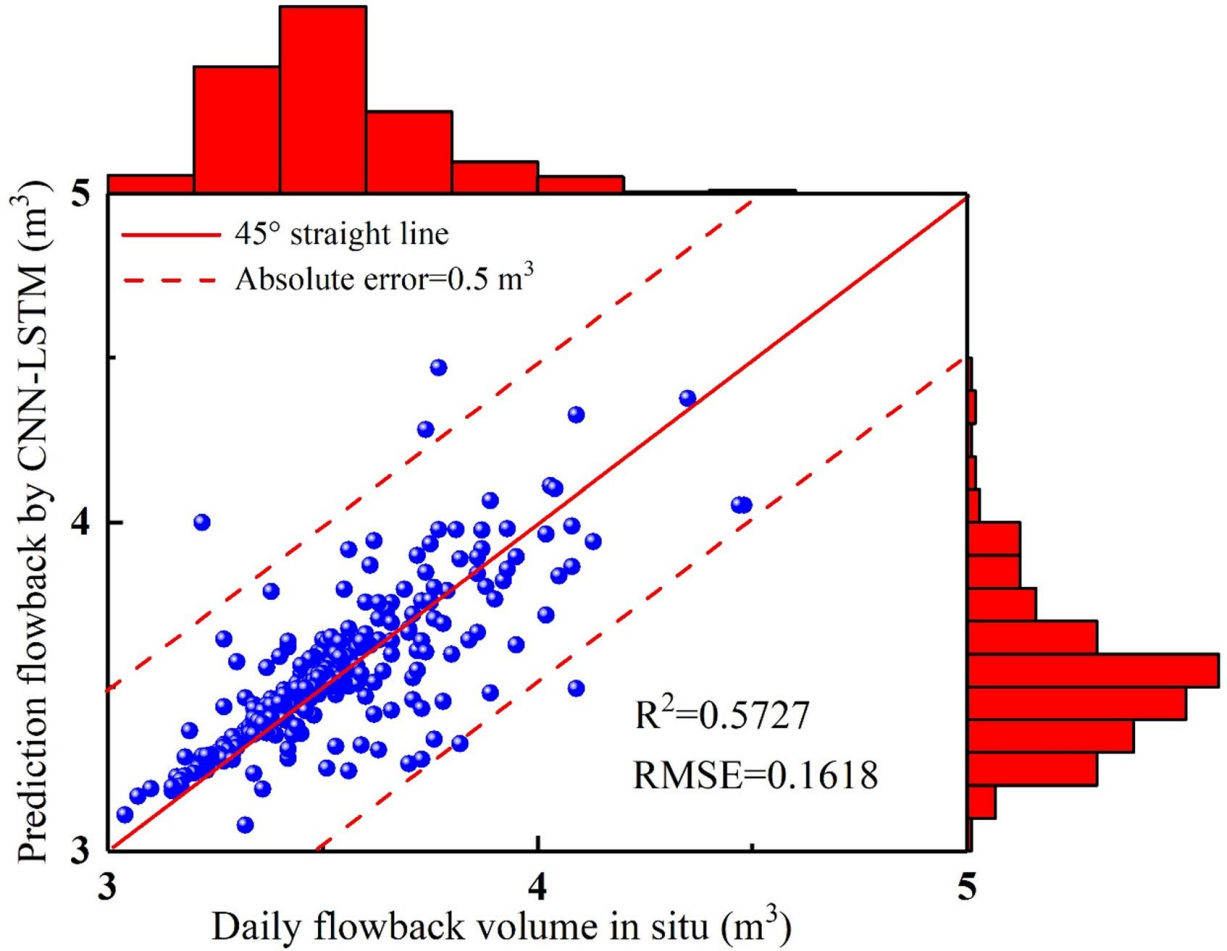
The comparison diagram of fracturing fluid flowback between CNN-LSTM and in-situ data.

In 1–17 days, the overall trend between predicted results and in-situ flowback data is consistent (Fig. 5–6). The CNN-LSTM’ values are generally higher than the in-situ data. In 18–33 days, the trend fluctuations between the CNN-LSTM’ values and the in-situ data are significant (Figs. 5 and 6). In 34–60 days, the in-site fracturing fluid flowback volume shows a continuous declining trend. The CNN-LSTM model captures this characteristic, and the predicted values also exhibit the same trend (Figs. 5 and 6). However, the CNN-LSTM’ values are still generally higher than the actual values. In 62–72 days, the in-site fracturing fluid flowback volume initially shows a slow decline, followed by a sharp decrease. Although the overall trend of the CNN-LSTM’ values align with the in-situ data, the model’s predictions for this complex temporal dynamic are clearly not ideal (Figs. 5 and 6). In 73–79 days, the overall trend of fracturing fluid flowback volume shows an increase, with some fluctuations (Figs. 5 and 6). The trend in the predicted results aligns with the actual flowback volume and exhibits similar fluctuations. In 80–119 days, the in-site fracturing fluid flowback volume exhibits significant fluctuations, with repeated increases and decreases. This presents considerable challenges for the CNN-LSTM model’s predictions, resulting in a substantial decline in accuracy. In 120–167 days, the in-site fracturing fluid flowback volume shows a continuous declining trend. The CNN-LSTM model accurately captures this distinct characteristic, with the CNN-LSTM’ values slightly higher than the in-situ data (Figs. 5 and 6). In 168–183 days, the in-site fracturing fluid flowback volume fluctuated up and down. The prediction accuracy obtained by CNN-LSTM is insufficient for this complex dynamic situation, resulting in a significant difference between the CNN-LSTM’ values and the in-situ data (Figs. 5 and 6). In 184–199 days, the in-site flowback volume shows a continuous slow decline. The CNN-LSTM predicted values match the actual values well. In 200–218 days, the in-site flowback volume first increases and then decreases. The CNN-LSTM’ values align with the in-situ data, with only slight overestimation at a few points. In 219–249 days, the in-site flowback volume first increases, then decreases, followed by another increase and decrease. The CNN-LSTM’ values follow the same trend as the in-situ data, and the data aligns well. In 250–281 days, the on-site fracturing fluid flowback volume shows slight fluctuations, with the CNN-LSTM’ values being slightly higher than the in-situ data (Figs. 5 and 6). To evaluate the accuracy of the CNN-LSTM predicted values compared to the in-situ data, two evaluation metrics, R² and RMSE, were used (Fig. 7). The results show that R^2^ = 0.5727 and RMSE = 0.1618 (Fig. 7), indicating that the prediction accuracy of the CNN-LSTM model is relatively good, but it has not yet reached the ideal level.

#### Fracturing fluid flowback predicted by CNN-GRU-AM

**Fig. 8:**
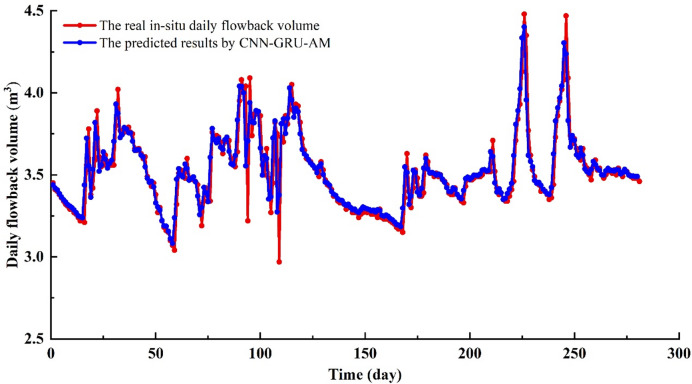
The fracturing fluid flowback predicted results by CNN-GRU-AM vs. in-situ data.

**Fig. 9:**
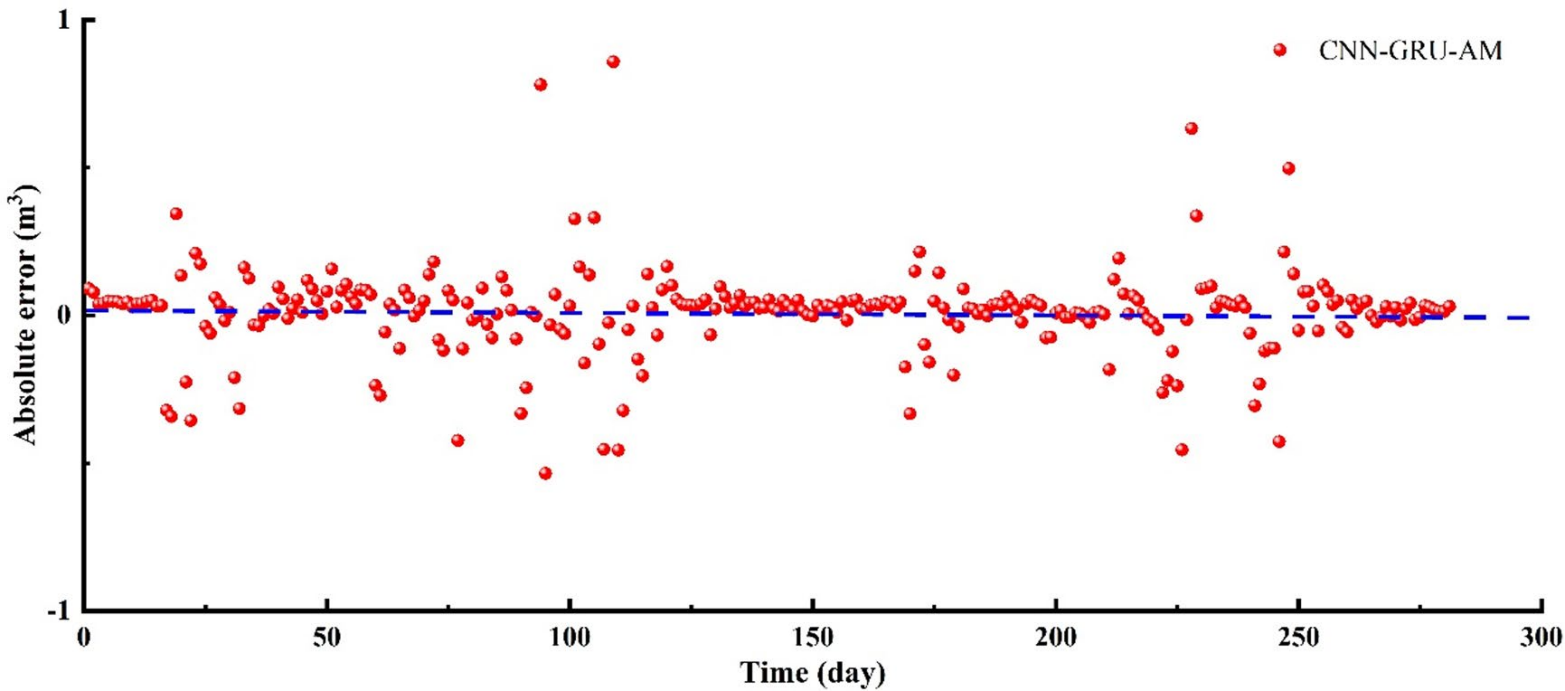
The absolute error of fracturing fluid flowback between CNN-GRU-AM and in-situ data.

**Fig. 10:**
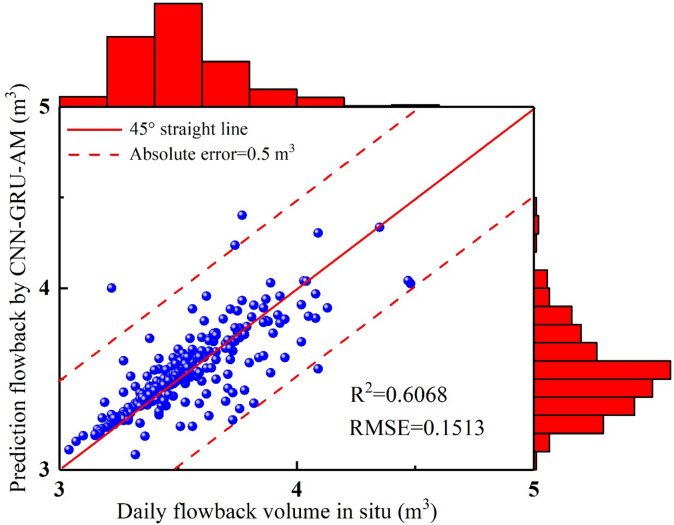
The comparison diagram of fracturing fluid flowback between CNN-GRU-AM and in-situ data.

In 0–17 days, the in-site fracturing fluid flowback volume continues to decline, and the model easily captures this simple characteristic (Fig. 8–9). The predicted trend aligns with the in-situ trend, with the CNN-GRU-AM’ values being slightly higher than the in-situ data. In 18–37 days, the amount of flowback fluid at the site shows fluctuations, initially rising and then falling, with this pattern repeating three times (Figs. 8 and 9). Due to the non-stationary nature of the time series data, the CNN-GRU-AM model’s prediction accuracy is significantly affected. As a result, there is a certain degree of error between the predicted values and the actual values, with the predicted values noticeably lower than the actual ones. In 38–59 days, the in-site fracturing fluid flowback volume shows a significant downward trend (Figs. 8 and 9). The CNN-GRU-AM model has a considerable advantage in short-distance learning, making it easier to obtain more global information, thereby improving the model’s accuracy. The CNN-GRU-AM’ values are higher than the in-site data. In 60–79 days, the in-site flowback volume exhibits significant fluctuations. The CNN-GRU-AM model’s prediction accuracy is notably insufficient for this situation, resulting in a large discrepancy between the CNN-GRU-AM model and in-site data (Figs. 8 and 9). In 80–116 days, the fracturing fluid is in an unstable flow stage, with significant fluctuations in the in-site fracturing fluid flowback data (Figs. 8 and 9). This stage exhibits the most severe fluctuations throughout the entire prediction process, making it the most challenging for the CNN-GRU-AM model to predict and result in the lowest accuracy. During this stage, a significant gap emerges between the CNN-GRU-AM model and in-situ data. In 117–169 days, the in-site fracturing fluid flowback shows a monotonous decline (Figs. 8 and 9). Given this simple characteristic, the CNN-GRU-AM model can easily capture it, with the predicted values being slightly higher than the actual values. In 170–182 days, the in-site flowback exhibited a trend of continuous increase followed by a decrease. Given this pronounced fluctuation feature, the CNN-GRU-AM predictive performance is evidently suboptimal, making it difficult to distinguish between the predicted values and the actual values. In 183–211 days, the in-site flowback volume first decreases and then gradually increases (Figs. 8 and 9). The CNN-GRU-AM model can easily capture this pattern, with predicted values being higher than actual values during the decline phase and lower than actual values during the increase phase. In 212–246 days, the in-site flowback volume briefly decreases before rapidly increasing to the highest point of the entire flowback stage. It then decreases again, followed by another rise to the peak value (Figs. 8 and 9). Although this stage exhibits significant fluctuations, the overall trend of the CNN-GRU-AM’ values and in-situ data remain consistent. However, the discrepancy between the CNN-GRU-AM’ values and in-situ data is relatively large. In 247–281 days, the in-site flowback shows a downward trend, with noticeable minor fluctuations in the latter half causing the curve to be uneven. The CNN-GRU-AM’values are higher than the in-situ data (Figs. 8 and 9). The R² between the CNN-GRU-AM model and in-situ for the is 0.6068, and the RMSE is 0.1513 (Fig. 10), suggesting that this model performs better than the CNN-LSTM model. However, its predictive performance has still not reached the optimal level.

#### Fracturing fluid flowback predicted by CNN-Transformer

**Fig. 11:**
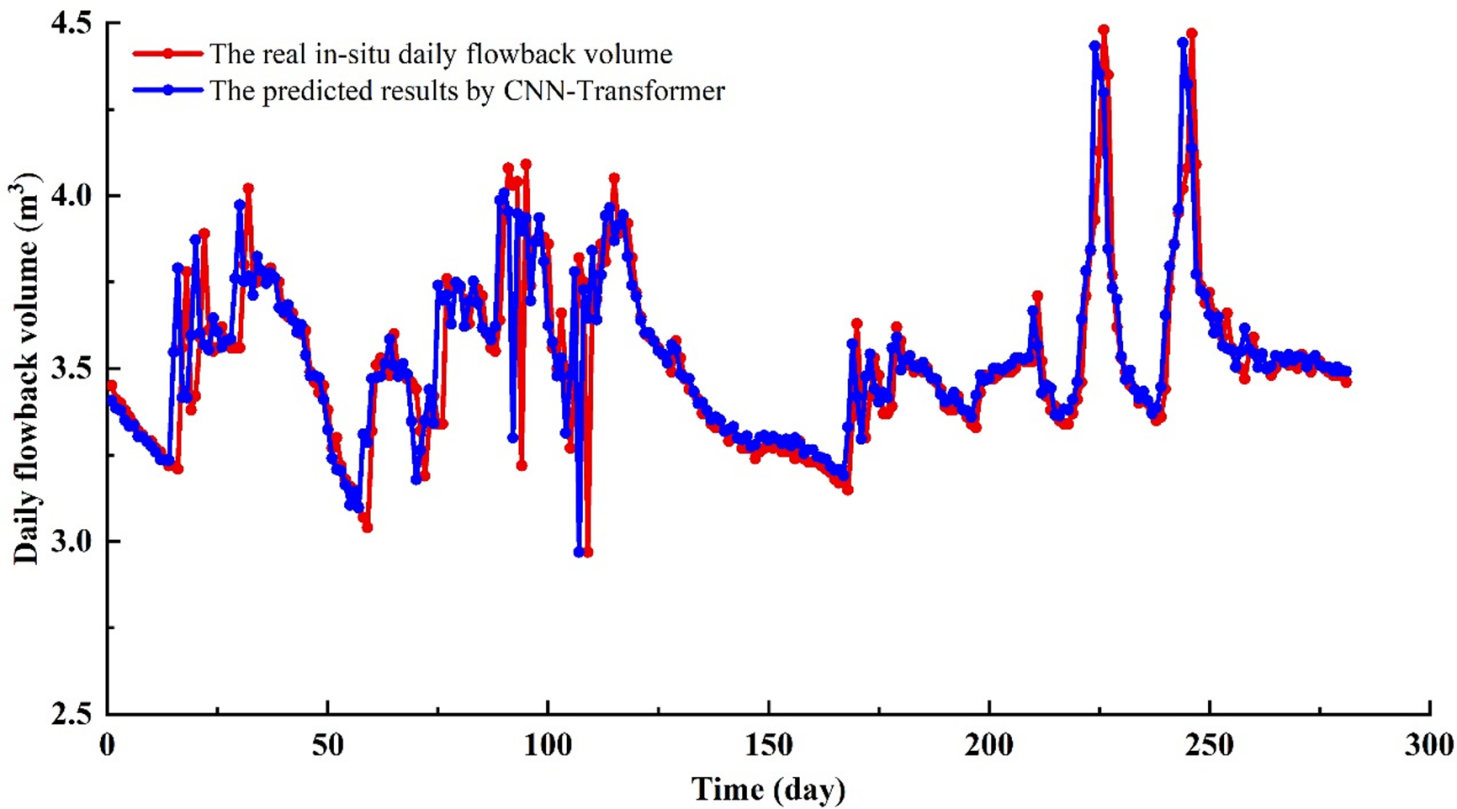
The fracturing fluid flowback predicted results by CNN-Transformer vs. in-situ data.

**Fig. 12:**
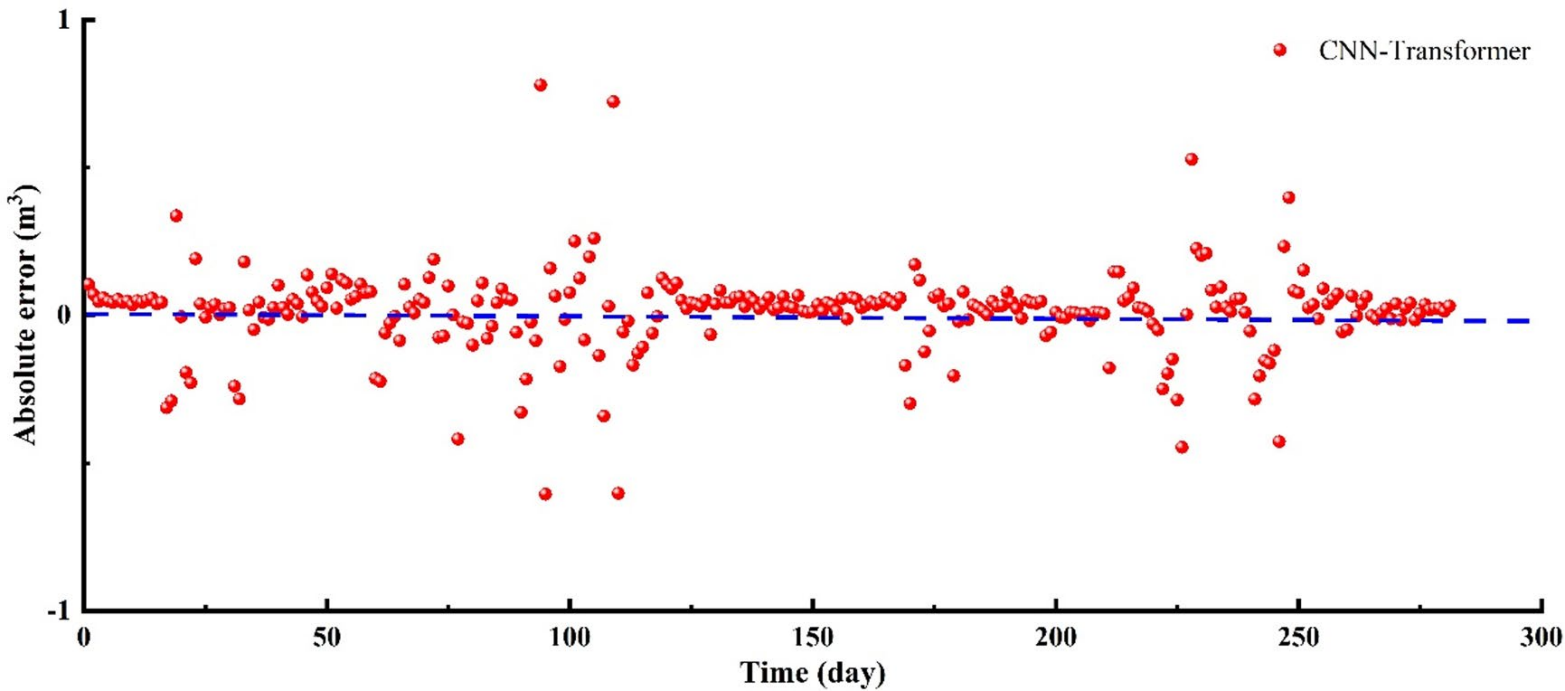
The absolute error of fracturing fluid flowback between CNN-Transformer and in-situ data.

**Fig. 13:**
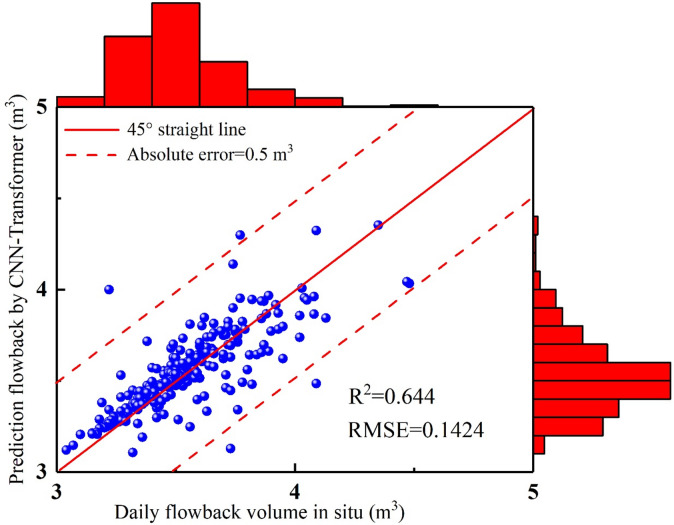
The comparison diagram of fracturing fluid flowback between CNN-Transformer and in-situ data.

In 1–17 days, the fracturing fluid in-situ data shows a continuous decline in the flowback volume (Fig. 11–12), which is a straightforward characteristic that the model can easily capture. The CNN-Transformer’ values closely match the in-situ data, with the trends also aligning well. In 18–32 days, the field flowback data shows continuous fluctuations, with an initial increase followed by a decrease, and this pattern occurred three times (Figs. 11 and 12). Despite the relatively complex time dynamics, the CNN-Transformer’ values closely match the in-situ data, with only minor errors at a few points. In 33–59 days, the field data of fracturing fluid flowback shows a continuous decline with slight fluctuations (Figs. 11 and 12). Given this straightforward temporal characteristic, the model effectively extracts features from the time series data, resulting in predicted values that closely match the actual values. In 60–72 days, the flowback field data first increases, followed by a brief stable phase, and then decreases with slight fluctuations during this stage (Figs. 11 and 12). The predicted values are in close agreement with the actual data. In 73–117 days, during the flowback process, as the fluid filtrates into the reservoir, the reservoir pressure increases. This stage is the most complex part of the flowback process and presents the greatest challenge for all predictive models. The lack of clear flowback patterns in this phase leads to a decrease in model accuracy. However, by effectively combining the advantages of specialized convolution operations with long-range dependency learning, the model successfully extracts key features, resulting in predicted values that closely match the actual data. This is also where the CNN-Transformer model outperforms other predictive models (CNN-LSTM, CNN-GRU-AM). In 119–169 days, the flowback volume in field shows a monotonic decrease (Figs. 11 and 12), primarily because the higher the viscosity of the produced fluid, the smaller the flowback volume, and the greater the bottomhole pressure. This is due to the increased flow resistance in the wellbore caused by the higher viscosity of the produced fluid, resulting in reduced flowback rates. Given this straightforward trend, the model’s predicted values show very little error compared to the actual values. In 170–198 days, the flowback volume shows an overall pattern of rising initially, followed by a decline, with distinct fluctuations in the middle (Figs. 11 and 12). The CNN-Transformer’ values closely match the in-situ data, with minimal errors, although the predictions are slightly higher than the actual values in the latter part of the process. In 199–253 days, after a brief increase, the fracturing fluid flowback volume begins to decrease, followed by a sharp rise, peaking, and then rapidly dropping (Figs. 11 and 12). The flowback volume then rises sharply to another peak before again declining rapidly, creating two distinct spikes in the data. Despite these fluctuations, the model effectively captures this fundamental pattern, with predicted values closely aligning with the actual data. In 254–281 days, the fracturing fluid flowback volume shows a stable trend with minor fluctuations, and the predicted values closely match the in-situ data with minimal error (Figs. 11 and 12). The CNN-Transformer model has an R^2^ of 0.644 and an RMSE of 0.1424 (Fig. 13), indicating that the model can effectively capture complex patterns and non-linear relationships in the data. Additionally, it captures temporal dependencies, enhancing the predictability of the original time series (Fig. 11 – 13). As a result, it can effectively analyze trend and cyclical components in the time series while preserving the sequential information of the original data to the greatest extent possible (Fig. 11 –13).

### Production prediction results

#### Production predicted by CNN-LSTM

**Fig. 14:**
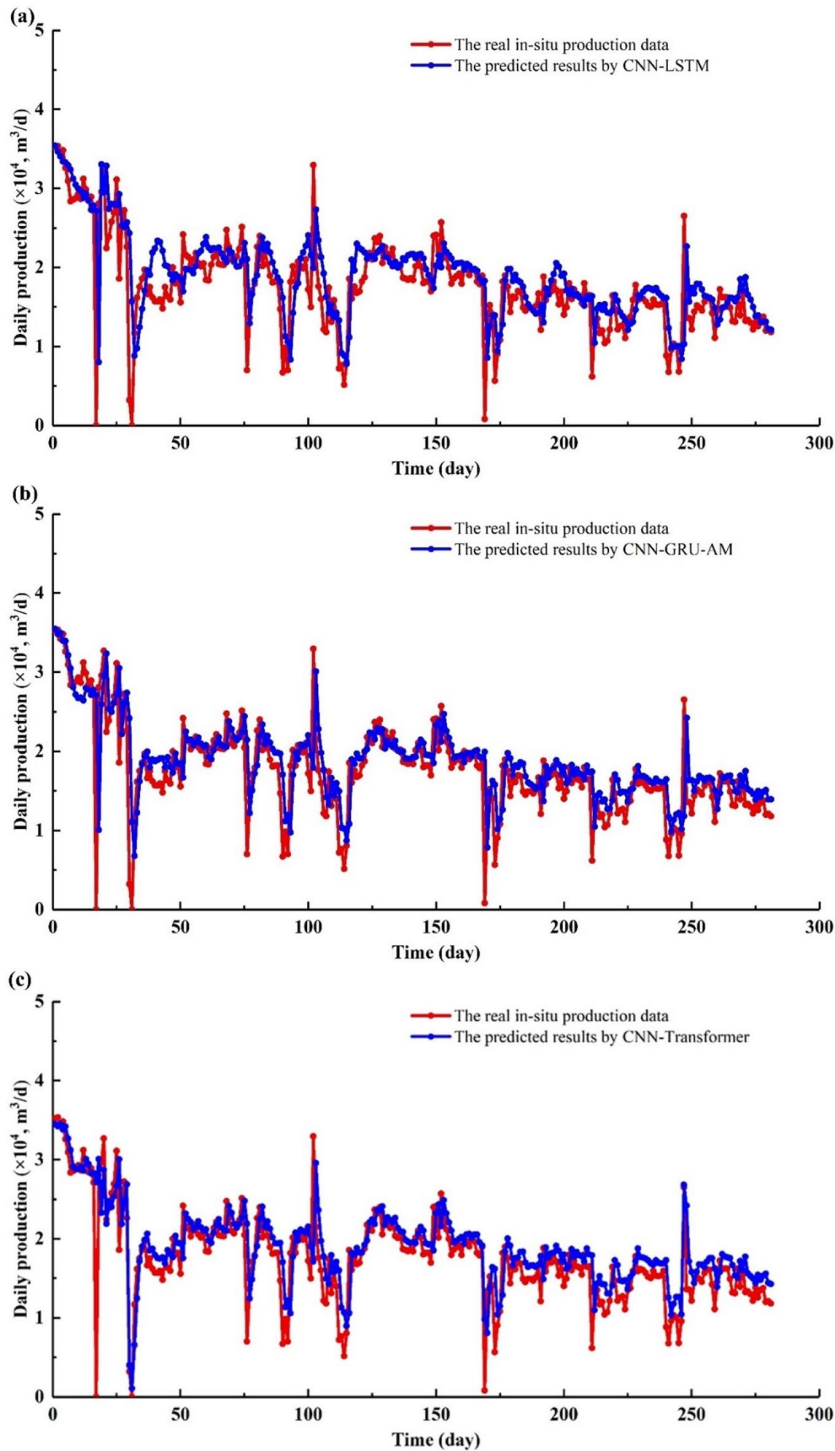
Daily gas production predicted results by different model’s vs. in-situ data. (**a**) CNN-LSTM; (**b**) CNN-GRU-AM; (**c**) CNN-Transformer.

**Fig. 15:**
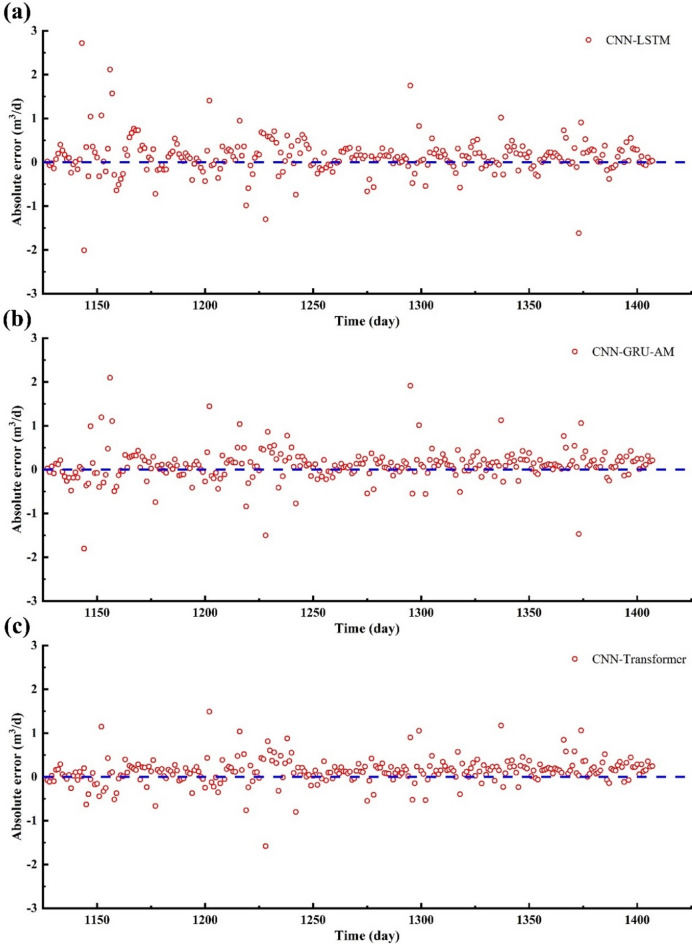
The absolute error of gas production between different model’s vs. in-situ data. (**a**) CNN-LSTM; (**b**) CNN-GRU-AM; (**c**) CNN-Transformer.

Figure 14a shows a comparison between the actual production data and the results predicted by the CNN-LSTM model. The actual production data and the predicted data show significant fluctuations in the initial stage, with the actual shale gas production data exhibiting several notable peaks and troughs within the 0–50 days (Fig. 14a−15a). In the 4–11 days, the actual production curve shows a trend of initially decreasing and then increasing. However, the trend predicted by the CNN-LSTM model is consistently decreasing (Fig. 14a−15a). In 21–24 days, the actual and predicted values exhibit the same trend, but the CNN-LSTM’ values are significantly higher than the in-situ data. In 26–31 days, the overall trend of the in-situ data and the CNN-LSTM’ values is consistent, but the CNN-LSTM’ trend is delayed (Fig. 14a−15a). The CNN-LSTM’ values are slightly higher than the in-situ data. In 38–47 days, the actual production shows a trend of initially decreasing and then increasing. However, the predicted production shows a trend of initially increasing and then decreasing (Fig. 14a−15a). In 51–56 days, the predicted production is significantly lower than the actual production, and their trends are also different. In 58–62 days, the actual production shows a clear trend of decreasing and then increasing, while the predicted production exhibits the opposite pattern (Fig. 14a−15a). In 82–92 days, both the predicted production and the actual production show a continuous downward trend, with the CNN-LSTM’ production being slightly higher than the actual values (Fig. 14a−15a). In contrast, in 93–96 days, the predicted production is lower than the in-situ data. In 126–132 days, the actual values show a continuous upward trend, while the predicted production initially rises and then falls (Fig. 14a−15a). The predicted values are higher than the in-situ data. The error between the CNN-LSTM’ production and in-situ data is relatively large. In 178–186 days, the predicted values were higher overall than the actual production (Fig. 14a−15a). The actual production shows a trend of initially decreasing and then increasing, while the predicted values show a trend of initially increasing and then decreasing. In 195–201 days, the predicted value is higher than the actual value, and it rises initially before declining, whereas the actual value consistently decreases (Fig. 14a−15a). In 213–224 days, the CNN-LSTM’ production is significantly higher than the in-situ data, and their trends are opposite to each other (Fig. 14a−15a). In 232–241 days, the predicted value is notably higher than the actual value. The predicted value first rises and then falls, while the actual value first falls and then rises. In 248–254 days, the predicted value is higher than the actual value, yet the overall trend of both the CNN-LSTM’ production and in-situ data remain the same (Fig. 14a−15a). In 269–274 days, the CNN-LSTM’ production is significantly higher than the actual value, and both the predicted and actual values show a downward trend (Fig. 14a−15a).

**Fig. 16:**
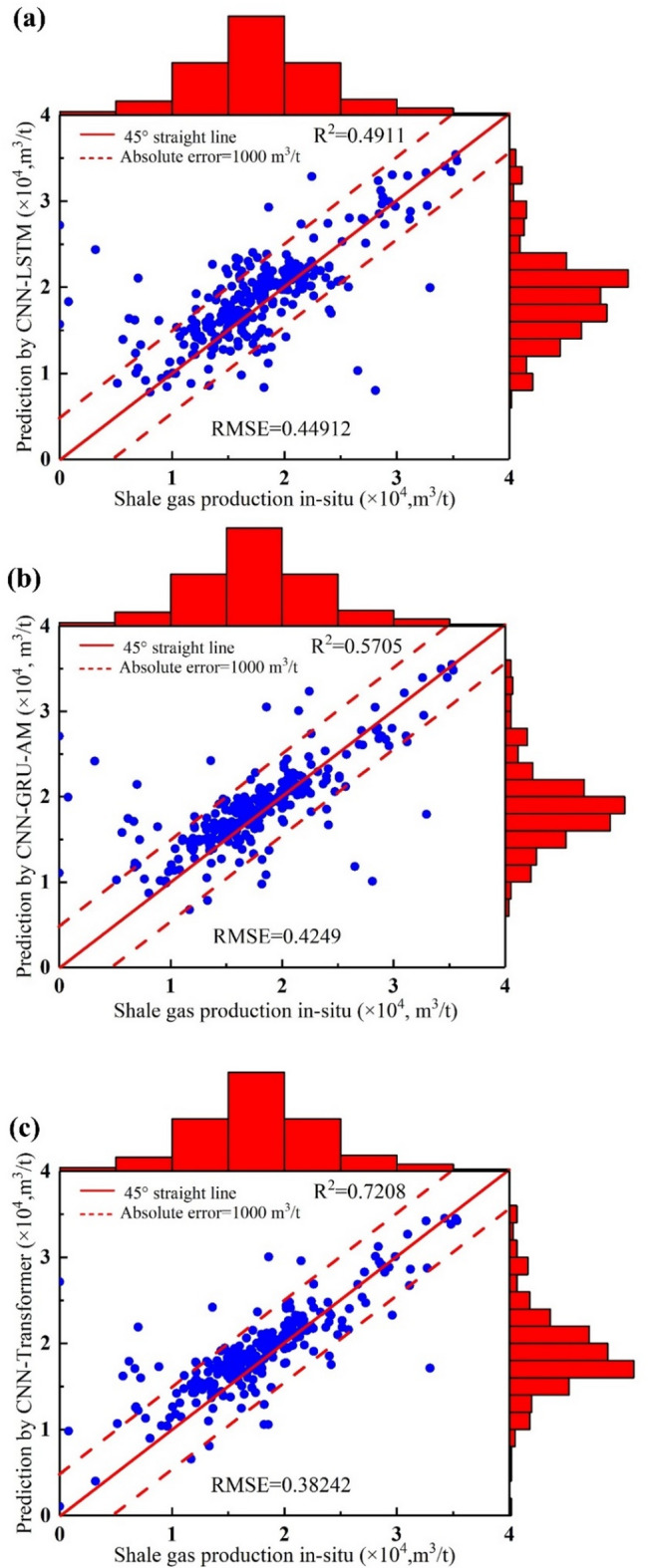
The comparison diagram of daily shale gas production between different model’s vs. in-situ data. (**a**) CNN-LSTM; (**b**) CNN-GRU-AM; (**c**) CNN-Transformer.

In Fig. 16a, the R^2^ and RMSE are 0.4911 and 0.44912, indicating the average error between the predicted and actual values. From the scatter plot, the data points roughly align along the 45-degree line, indicating a certain level of correlation between the CNN-LSTM’s production and the in-situ data (Fig. 16a). However, based on the R^2^ value, there is still significant room for improvement in the model’s predictive performance. Most of the data points are within the absolute error lines, indicating that most of the predicted errors are within an acceptable range. Based on the R^2^ and RMSE, the predicted performance of CNN-LSTM is inferior to that of CNN-Transformer (Fig. 16a).

**Fig. 17:**
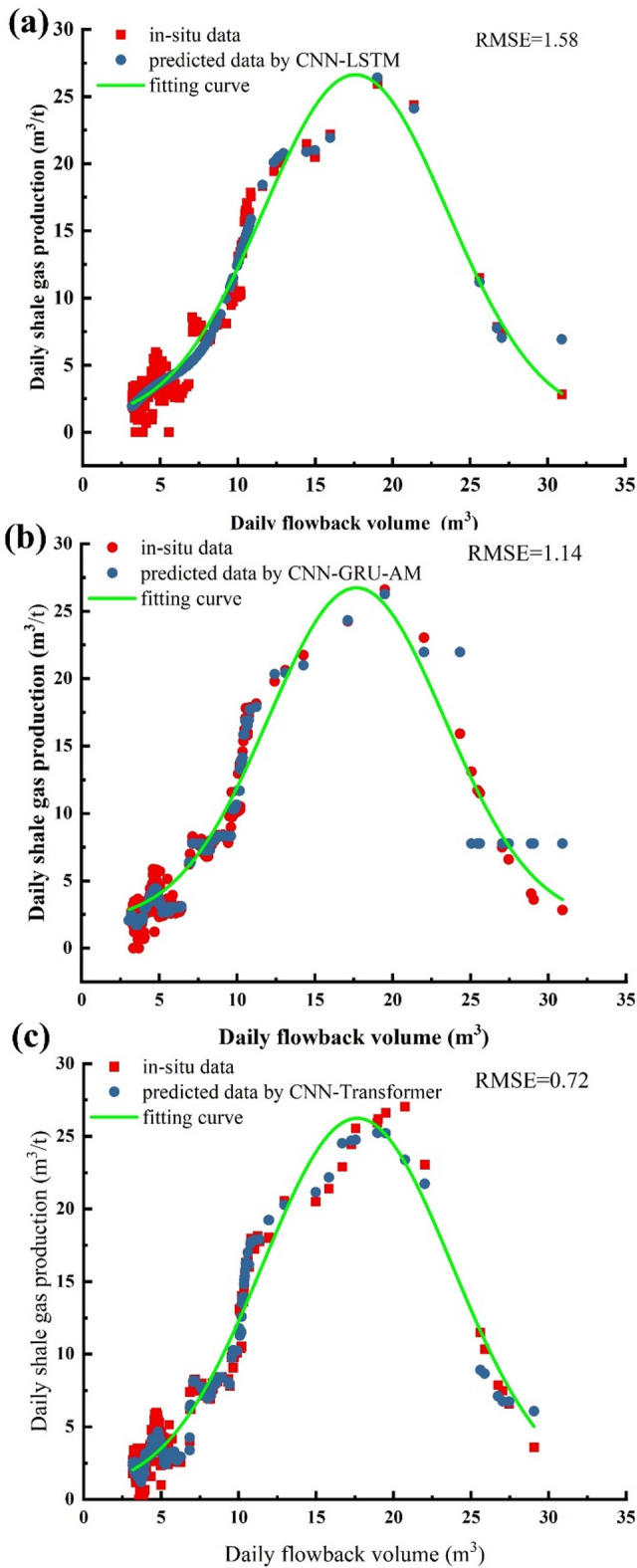
The fitted curve between fracturing fluid flowback and daily gas production between different model’s vs. in-situ data. (**a**) CNN-LSTM; (**b**) CNN-GRU-AM; (**c**) CNN-Transformer.

Figure 17a illustrates CNN-LSTM model to predict fracturing fluid flowback and daily production data. The prediction values show minimal error when compared to the actual values. Notably, the model made predictions using only 20% of the overall data. However, the latter portion of the data is relatively sparse, and although the errors are small, the predictive performance might decline with an increase in data volume. Additionally, the fitted curve between the fracturing fluid volume and daily production adequately covers most of the data points. However, an RMSE of 1.58 indicates that the predictive performance of this model is inferior to that of the CNN-GRU-AM and CNN-Transformer models (Fig. 17a).

#### Production predicted by CNN-GRU-AM

Figure 14b displays a comparison of the shale gas production between the CNN-GRU-AM model and the in-situ data. In the 9–13 days, the predicted results are evidently lower than in-situ actual production data. In the 39–46 days, the predicted results are significantly higher than actual production data (Fig. 14b−15b). In the 84–96 days, the predicted results are evidently lower than actual production data, while the overall trend has been consistent (Fig. 14b−15b). In 126–128 days, the predicted results are lower than the in-situ actual production data. In 144–148 days, the predicted results are slightly higher than the actual production data (Fig. 14b−15b). In 175–189 days, the predicted results are higher than the in-situ actual production data, while the trend is similar. In 220–226 days, the predicted results are substantially higher than the in-situ actual production data, with the consistent fluctuation trend. In 248–259 days, the predicted results are higher than the in-situ actual production data. In 271–281 days, the predicted results are higher than the in-situ actual production data. Although the CNN-GRU-AM model captures the fluctuations in actual production data quite well, the predicted accuracy and reliability is not as good as CNN-Transformer (Fig. 14b−15b).

R^2^ value is 0.5705, suggesting the predictive performance of CNN-GRU-AM is not as good as that of CNN-Transformer (Fig. 16b). RMSE obtained by CNN-Transformer is 0.4249, a lower RMSE indicates better model performance (Fig. 16b). Most of the blue dots are close to line 45, indicating that the CNN-GRU-AM model’s predictions are reasonably accurate but not perfect (Fig. 16b). Some points lie outside the dashed red lines, indicating predictions with an absolute error greater than 1000 m^3^/t. This suggests areas where the model’s predictions significantly deviate from actual values (Fig. 16b). The histograms show the concentration of actual and predicted values. Both distributions are relatively similar but not identical, indicating that while the model captures the general trend, there are discrepancies in specific ranges (Fig. 16b). Figure 16b suggests that the CNN-GRU-AM model is reasonably effective at predicting shale gas production, with moderate correlation and some degree of error.

The CNN-GRU-AM model was used to fit the data of fracturing fluid flowback volume and daily gas production (Fig. 17b). As shown in Fig. 17b, the prediction values obtained by the model have a small error compared to the actual values. However, there is a significant discrepancy between the CNN-GRU-AM’ value and in-situ data at the last few data points (Fig. 17b), indicating that the accuracy is lower than that of the CNN-Transformer model. While the fitted curve covers most of the data points relatively well, the RMSE value of the model is higher than that of the CNN-Transformer model (Fig. 17b).

#### Production predicted by CNN-Transformer

Figure 14c presents the shale gas production prediction by the CNN-Transformer. CNN-Transformer models have demonstrated clear advantages in handling time series problems, owing to their capacity to capture nonlinear and intricate relationships within sequential data. In contrast, CNN-GRU-AM and CNN-LSTM use a recursive approach for each step’s prediction, causing errors to gradually accumulate.

Figure 14c shows the comparison between the shale gas production predicted by the CNN-Transformer model and the in-situ production data. The overall trend of the shale gas production precited by CNN-Transformer and in-site production is very close, indicating that the CNN-Transformer model performs well in capturing the fluctuations of the actual production data. Both curves show high volatility, reflecting the natural variation in shale gas production (Fig. 14c). In the first 50 days, the actual production data exhibits significant fluctuations, and the predicted data follows these fluctuations closely (Fig. 14c). From 50 to 150 days, both the actual production data and the predicted data show frequent fluctuations, and the match between the two is high (Fig. 14c). From 150 to 250 days, the predicted data still closely follows the actual data, although there are some discrepancies at certain peaks and troughs (Fig. 14c). Throughout the entire period, the actual production data and predicted data obtained by CNN-Transformer are basically in sync, demonstrating the effectiveness of the prediction model. The CNN-Transformer model can predict actual production data well in most cases, reflecting its good capability in capturing complex time series data. Although the two curves overlap well for the most part, there are some deviations between the CNN-Transformer model’s value and the in-situ data at certain points (Fig. 14c). This could be due to the CNN-Transformer model’s slightly inferior performance in handling extreme or anomalous values. Overall, the CNN-Transformer model performs excellently in predicting shale gas production, accurately reproducing the fluctuation trends of the actual production data.

Figure 15c shows the relative error between the shale gas production predicted by the CNN-Transformer model and the actual production data. Red circles represent the relative error data points of the CNN-Transformer model. Blue dashed line is the zero level of relative error. The distribution of the red circles is relatively concentrated around the zero-level line, indicating that the prediction error of the CNN-Transformer model is generally small (Fig. 15c). From 1150 days to 1400 days, the red circles are mainly distributed between − 2% and 2%, indicating that most errors are small (Fig. 15c). The error data points are evenly distributed over the entire period, with no significant trend bias, demonstrating stable error performance of the model at different time points (Fig. 15c). The model can accurately predict actual production data in most cases, reflecting its superiority in handling complex time series data. Although there are a few discrete error points, the overall error magnitude is not large, indicating high reliability and practical value of the model in in-situ shale gas applications (Fig. 15c).

Figure 16c shows the comparison between the shale gas production predicted by the CNN-Transformer model and the actual production. Each blue dot represents a pair of predicted and actual values. The red histograms at the top and right side show the distribution of actual and predicted production values. Figure 16c shows an R² value of 0.7208, demonstrating a strong alignment between the model’s predictions and the in-situ data. Figure 16c shows an RMSE value of 0.38242, indicating the average error between the predicted and actual values. Most blue dots are distributed near the 45-degree line, indicating that the predicted values are close to the actual values. The red histograms at the top and right show similar distributions for actual and predicted production values, further validating the accuracy of the model’s predictions. The CNN-Transformer model performs well in predicting shale gas production, with a high R² value indicating strong explanatory power and the ability to capture most of the variation in the actual production data. The low RMSE value indicates small prediction errors, demonstrating high prediction accuracy. Both the scatter plot and histograms show similar distributions between predicted and actual values, indicating high reliability of the model.

To more accurately establish the relationship between fracturing fluid flowback volume and daily gas production, a CNN-Transformer model was employed to predict data spanning 1406 days of flowback volume and daily production. Subsequently, the data was fitted to a curve. In Fig. 17c, the flowback volume is represented on the x-axis, while daily production is plotted on the y-axis. Figure 17c indicates that the predicted values closely align with the in-situ data, with only minor discrepancies in a few days. Additionally, the fitted curve exhibits a strong correspondence with the actual data points.

The CNN-Transformer model’s marked reduction in analysis error for shale gas production prediction stems from its hybrid architecture, which synergistically combines the strengths of CNNs for local feature extraction with the Transformer’s global attention mechanism for capturing long-range temporal dependencies. This design addresses key limitations in shale gas time-series data, such as nonlinearity, noise from environmental disruptions (well shutdowns or weather events), and the complex coupling between fracturing fluid flowback and production dynamics. In contrast, CNN-LSTM and CNN-GRU-AM rely on recurrent structures (LSTM/GRU), which propagate errors sequentially and struggle with long-term dependencies due to vanishing gradients or computational inefficiency.

1. Enhanced Capture of Spatiotemporal Dependencies. CNNs in the hybrid framework use 1D causal convolutions (with dilated kernels) to extract local correlations in irregular observational data, such as short-term fluctuations in production and flowback sequences. This provides robust sequence embeddings that preserve temporal locality without future leakage. The Transformer encoder-decoder then applies MSA and MIA to model global interactions across historical and future time steps. This allows dynamic handling of nonlinear path dependencies in gas-water two-phase flow, extracting state information from prior frames. As a result, the model better captures the negative correlation between flowback rate and long-term production, reducing error accumulation in volatile periods. In comparison, CNN-LSTM and CNN-GRU-AM feed CNN-extracted features into recurrent gates (forget/input/update), which process sequences step-by-step. This recursive nature leads to gradual error propagation, particularly in non-stationary data with outliers (equipment maintenance), resulting in higher RMSE and lower R2.
2. Improved Robustness to Noise and Irregularity. The attention mechanisms enable data fusion across multi-scale features, enhancing resilience to noise interference and environmental disturbances—common in shale gas datasets (sensor errors or shut-in periods). Skip connections and LN in the encoder-decoder further stabilize training, preventing overfitting on sparse or high-dimensional inputs. RNN-based models like LSTM/GRU-AM are temporally constrained, often underperforming on long sequences (1406 days in dataset) due to limited memory of distant events. This is evident in their poorer handling of trend shifts (days 82–92 in production prediction), leading to larger deviations.
3. Efficiency in Parallel Processing. Transformer’s parallelizable structure accelerates convergence on large datasets, allowing better optimization of hyperparameters (via 10-fold cross-validation). This yields higher generalization, as validated by our K-fold metrics, where CNN-Transformer consistently outperforms baselines across folds. Empirical evidence from our results supports this: The CNN-Transformer closely tracks production fluctuations, with errors concentrated near zero and strong alignment in scatter plots. The fitted curve between flowback and production further illustrates its ability to reveal underlying mechanisms, such as reduced long-term production with higher flowback rates.

## Conclusion

Based on the shale gas production data and fracturing fluid flowback data from the Zhaotong shale gas demonstration area on the periphery of the Sichuan Basin, this paper constructs a CNN-Transformer model to robustly predict gas production and fracturing fluid flowback, establishing a mathematical relationship between gas production and fracturing fluid flowback.

The CNN-Transformer model can extract temporal features that capture long-term dependencies in time series data. By utilizing a parallel structure, it accelerates model training and inference, and better captures features in temporal sequence data. This allows it to model time series with complex temporal dynamics effectively.

CNN-Transformer model can effectively improve predicted precision of shale gas production and flowback volume data without substantially compromising the performance of AI models, especially when applied to larger datasets. A corresponding benchmark analysis showed that the CNN-Transformer model significantly outperforms the CNN-LSTM and CNN-GRU-AM models in shale gas production and fracturing fluid flowback prediction accuracy.

The findings are promising and suggest that CNN-Transformer model could become standard practice in developing AI models for shale gas, helping to establish the mathematical relationship between shale gas production and fluid flowback volume while leveraging the power of AI for better prediction.

The implications of this research are profound for shale gas production and flowback volume prediction. By validating that CNN-Transformer model can be effectively applied to predict production and flowback volume without rendering the data and AI models unusable, this paper pave the way for more widespread adoption of CNN-Transformer model in petroleum field.

## Supplementary Information

Below is the link to the electronic supplementary material.

Supplementary Material 1

## Data Availability

The datasets used and/or analyzed during the current study are available from the corresponding author on reasonable request.
