# Supplementary material for: Exploring the predictive performance of deep learning for fracturing fluid flowback and shale gas production
Source: Sci Rep. 2025 Nov 28;15:42748. doi: 10.1038/s41598-025-26761-z (PMC12663573; doi:10.1038/s41598-025-26761-z)
Supplement: Supplementary file 1 — Supplementary Material 1 [file 41598_2025_26761_MOESM1_ESM.docx]

**Appendix A: Detailed Methodology**

## A.1 The architecture of CNN, LSTM and GRU

### A.1.1 The architecture of CNN

CNN is a type of deep feedforward neural network that centers around convolution operations[23]. The feature extraction of data in a CNN mainly relies on the convolutional kernels in the convolutional layers (Figure A1)[24]. The main processing steps in the input layer involve mean subtraction, normalization, and principal component analysis (Figure A1). The convolutional layer is the most important layer in a convolutional neural network[24]. In this convolutional layer, there are two key operations: local connectivity and sliding windows (Figure A1). Each neuron is treated as a filter, and a window, also known as a receptive field, slides over the data, performing calculations with the filter on the local data. The formula for calculating the size of the output of any given convolutional layer is[4]:

*O*=$\frac{(W-K+2P)}{S}$+1 (1)

where *O* is the output size, *K* is the filter size, *P* is the padding, and *S* is the stride.

In a convolutional layer, the non-linear layer, applies a non-linear mapping to the output of the convolutional layer (Figure A1). CNNs commonly use the ReLU as an activation function. ReLU has the characteristics of fast convergence and simple gradient computation. The pooling layer is typically inserted between consecutive convolutional layers in a CNN. The pooling layer commonly employs two main methods: max pooling and average pooling (Figure A1). The fully connected layer establishes weighted connections between all neurons of two consecutive layers. In a convolutional neural network, the fully connected layer is typically located at the end.


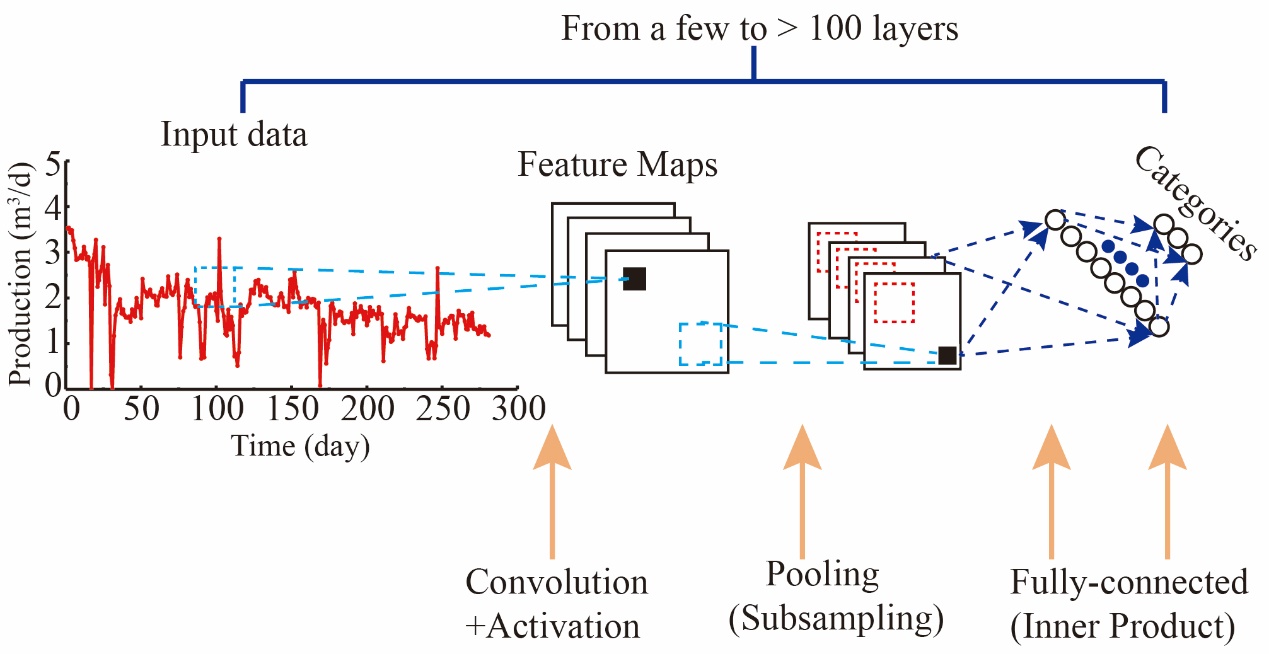
Figure A1. The structure of CNN.

### A.1.2 The architecture of LSTM


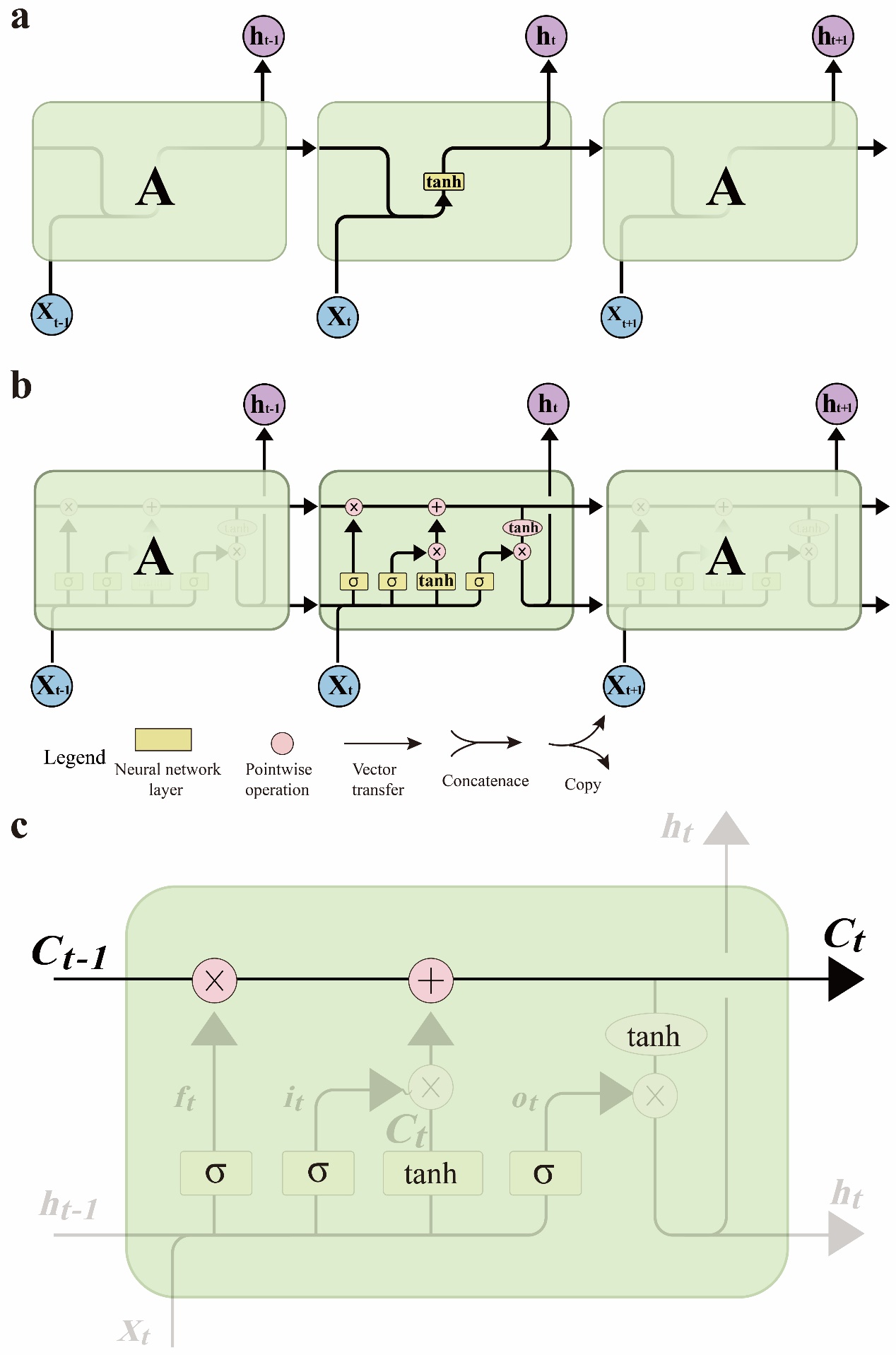


Figure A2. The basic framework of RNN and LSTM. (a) The basic framework in a standard RNN; (b) the four interacting layers in an LSTM; (c) the cell state in LSTM.

All RNNs have repeated neural network modules, which is a simple structure, such as a single tanh layer (Figure A2 (a)). LSTM also follows the chain-like structure of RNNs with repeated modules, but the repeating module in LSTM has a different structure[25]. Instead of a single neural network layer, it consists of four interacting components in a very special way (Figure A2 (b)). Neural network represents a single-layer neural network, that is, the operation of *w^T^x+b*. The difference lies in the activation functions used. *σ* represents the sigmoid function, which compresses the data into the range [0, 1][25]. *Tanh* refers to the hyperbolic tangent activation function, which normalizes the data into the range [-1, 1] (Figure A2(b)). Pointwise Operation refers to element-wise operations between two matrices (Figure A2(b)). If represented by the symbol×, each element in the resulting matrix is the product of the corresponding elements from the two matrices of the same dimensions. Vector transfer defines the matrix transfer. Concatenate is matrix concatenation, where two matrices are joined together without any computation. Copy represents one matrix becomes two identical copies (Figure A2(b)).

In the LSTM architecture, the connections between nodes are represented by black lines, which carry entire vectors from the output of one node to the input of another node (Figure A2 (c))[25]. The pink circles represent pointwise operations, such as vector addition, while the yellow rectangles represent learned neural network layers. The lines that are connected represent vector concatenation, where the information from different sources is combined into a single vector. The key component of the LSTM architecture is the cell state (Figure A2(c)), which runs horizontally across the top of the diagram[26]. The cell state serves as a linear pathway that runs through the LSTM module, allowing information to be preserved and carried over long sequences (Figure A2(c)).

The LSTM architecture incorporates specialized structures called "gates" to strategically retain or discard information to the cell state[27]. Each gate in an LSTM consists of two main components: a sigmoid neural network layer and a pointwise multiplication operation. The LSTM architecture has three gates that are used to protect and control the cell state: the forget gate, the input gate, and the output gate (Figure A3)[27]. In LSTM, the first step is to decide what information we're going to discard from the cell state[28]. This decision is made through a layer called the forget gate layer (Figure A3(a)). This gate reads *h_t-1_* and *x_t_* and outputs a value between 0 and 1 for each number in the cell state *C_t-1_*. The formula of forget gate is described as [28]:

(2)


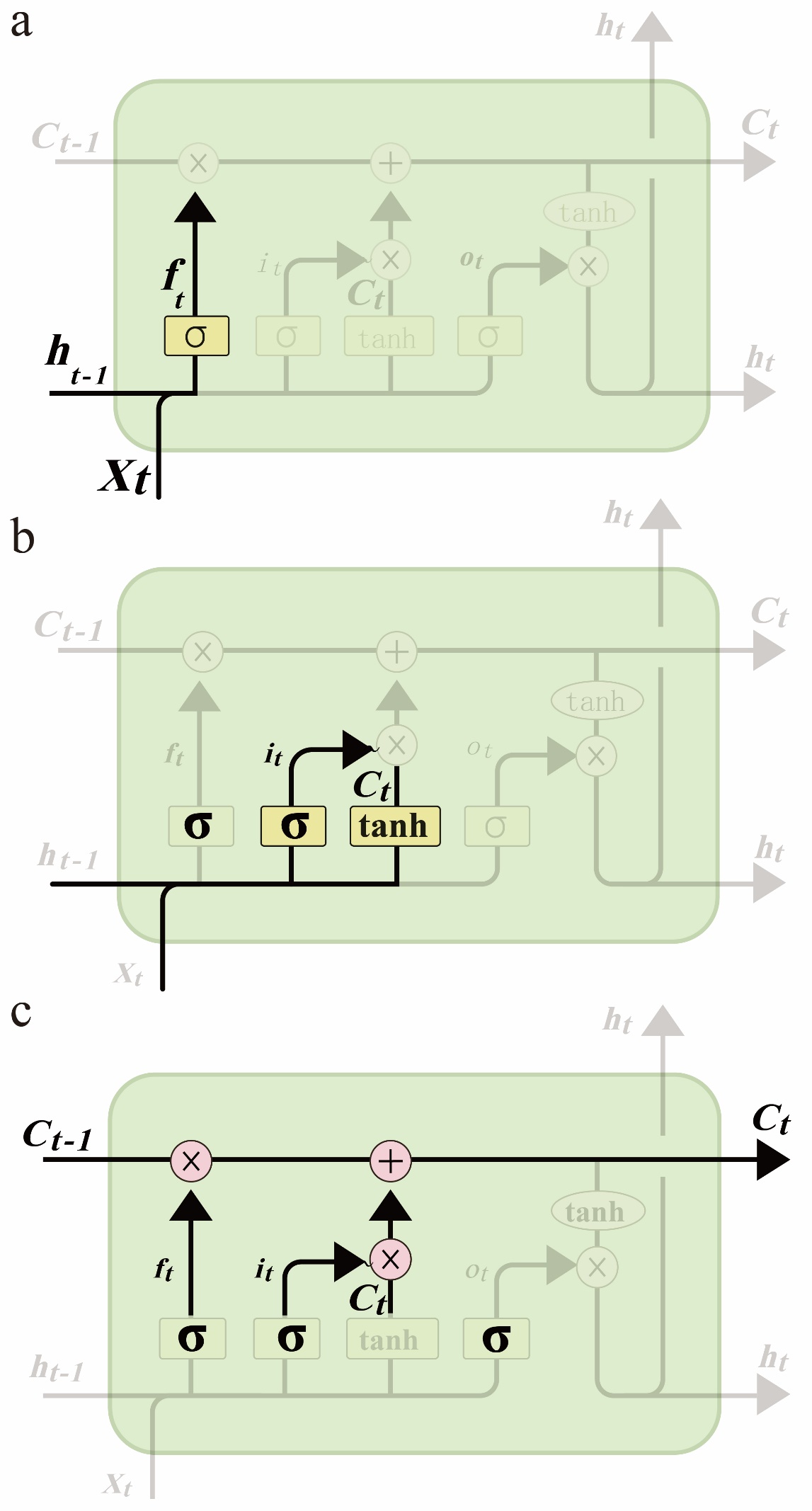


Figure A3. The forget gate, updating gate, and second operation of the update gate of LSTM. (a)

Forget gate of LSTM; (b) Updating gate operation; (c) The second operation of the update gate, update the C conveyor belt.

The next step is to determine what new information will be stored in the cell state (Figure A3(b))[28]. First, a sigmoid layer called the ‘input gate layer’ determines which values will update. Then, a *tanh* layer creates a new candidate value vector, which will be added to the state (Figure A3(b)). The basic formula of input gate is as follow[28]:

(3)

(4)

The update to the cell state *C_t_* is a key component of the LSTM model. It is calculated as follows[28]:

$C_{\text{t}}=f_{t}*C_{t-1}+i_{t}*\widetilde{C_{\text{t}}}$ (5)

where *f_t_* is the output of the forget gate, which determines how much of the previous cell state *C_t-1_* to "forget" or retain. *i_t_* is the output of the input gate, which determines how much of the new candidate cell state $\tilde{C}_{t}$ to incorporate into the new cell state *C_t_*. $\tilde{C}_{t}$is the new candidate cell state, which is computed based on the current input *x_t_* and previous hidden state *h_t-1_*.

The *f_t_*, given by the forget gate, multiplied by *C_t-1_* represents selective forgetting (retention) of past information (Figure A3(c))[28]. The new information $\tilde{C}_{t}$multiplied by *i_t_* represents selective forgetting (retention) of new information. Finally, adding these two parts together gives the new state *C_t_* (Figure A3(c)). This is the new candidate value, which varies according to the extent we decide to update each state.

The last part is the output of the LSTM (Figure 6(a)). The cell state *C_t_* has already been updated. *o_t_* is still passed through a sigmoid function to determine what to output, and *C_t_*, after being scaled by a *tanh* function, is multiplied by *o_t_*​. This constitutes the output for this timestep [28].

$o_{t}=\sigma(W_{o}[h_{t-1},x_{t}]+b_{o})$ (6)

$h_{t}=o_{t}*\tanh(C_{\text{t}})$ (7)

### A.1.3 The architecture of GRU

Cho et al. (2014) first introduced a typical GRU. The GRU which combines the forget gate and the input gate into a single update gate (Figure A4(b))[29]. It also merges the cell state and the hidden state, along with some other modifications. The GRU model employs only two gates: the update gate and reset gate (Figure A4(b))[4]. The specific architecture is illustrated in the figure below (Figure A4(b)).


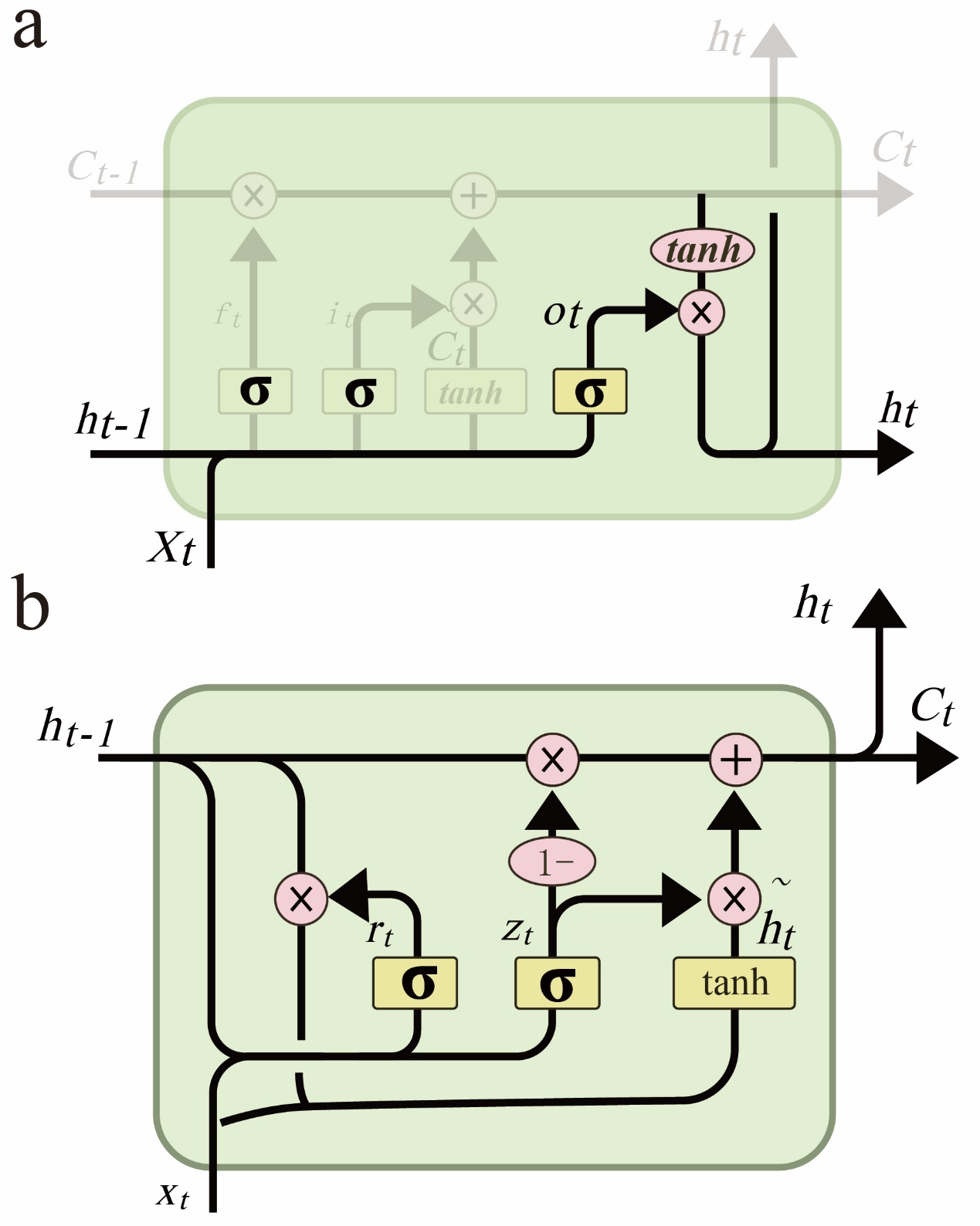
Figure A4. The output of LSTM and basic framework of GRU. (a) The output information of LSTM; (b) the basic framework of GRU.

First, introduce the reset gate (*r_t_*) and the update gate (*z_t_*) in GRU. The calculation methods are consistent with those used in the LSTM gates. The update gate controls the extent to which the previous time step's state information is incorporated into the current state[30]. The reset gate controls how much information from the previous state is written into the current candidate set. A smaller reset gate implies that less information from the previous state is written in[30].

$\text{r}_{t}=\sigma(W^{(r)}x_{t}+U^{(r)}h_{t-1})$ (8)

$\text{z}_{t}=\sigma(W^{(z)}x_{t}+U^{(z)}h_{t-1})$ (9)

*where U^(z)^*, *W^(z)^*, *U^(r)^*, *W^(r)^*, *U*, *W is* trainable parameter matrix; $\sigma$is the activation function of Sigmod.

Next is the computation of the candidate hidden layer$\mathop{h_{t}}\limits^{\text{～}}$, which is similar to the$\mathop{C_{t}}\limits^{\text{～}}$ in LSTM. This candidate hidden layer can be seen as the new information at the current time step. The parameter *r_t_* is used to control how much of the previous memory should be retained. The calculation formula is as follows[30]:

$\widetilde{h_{t}}=\tanh(r_{t}Uh_{t-1}+Wx_{t})$ (10)

Finally, *z_t_* controls how much information from the previous hidden layer *h_t-1_* needs to be forgotten and how much information from the current hidden layer $\mathop{h_{t}}\limits^{\text{～}}$ needs to be added. This results in *h_t_*, which is the final hidden layer output. It is important to note that the difference between GRU and LSTM here is that GRU does not have an output gate[30].

$h_{t}=(1-z_{t})\tilde{h}_{t}+z_{t}h_{t-1}$ (11)

where *z_t_* is the gate of renewal, *r_t_* is the reset gate, *h_t_* is the hidden layer input; $\widetilde{h_{t}}$ is aggregation of input *x_t_* and past hidden layer state *h_t-1_*;.

## A.2 The architecture of CNN-LSTM

The CNN-LSTM model consists of two parts (Figure A5). The first part is the CNN, which includes convolutional layers and max pooling[26]. It preprocesses the raw data and feeds it into the CNN convolutional layers. The convolutional layers adaptively extract spatial features using convolutional kernels. These layers traverse the input information, performing convolution operations between the weights of the convolutional kernels and local sequences to generate preliminary feature matrices. The max pooling layer takes the feature matrix obtained from the previous convolutional layer as input and slides a pooling window over this matrix. In each sliding step, it selects the maximum value within the pooling window and outputs a more expressive feature matrix.

After pooling, an LSTM layer is connected to extract relevant vectors constructed by the CNN as a long-term time series, which serves as the input data for the LSTM (Figure A5)[31]. The flattening layer flattens the output of the convolutional layer. In the model, a Flatten layer is added to compress the data into a one-dimensional array with a single dimension representing length, height, width, and channel[31]. To compress the features extracted by multiple convolutional feature extraction frameworks or fuse them from the output layer, a fully connected layer is used to aggregate the learned features. The activation function used is ReLU, which has a one-sided suppression effect and can introduce more sparsity, avoiding the vanishing gradient problem.


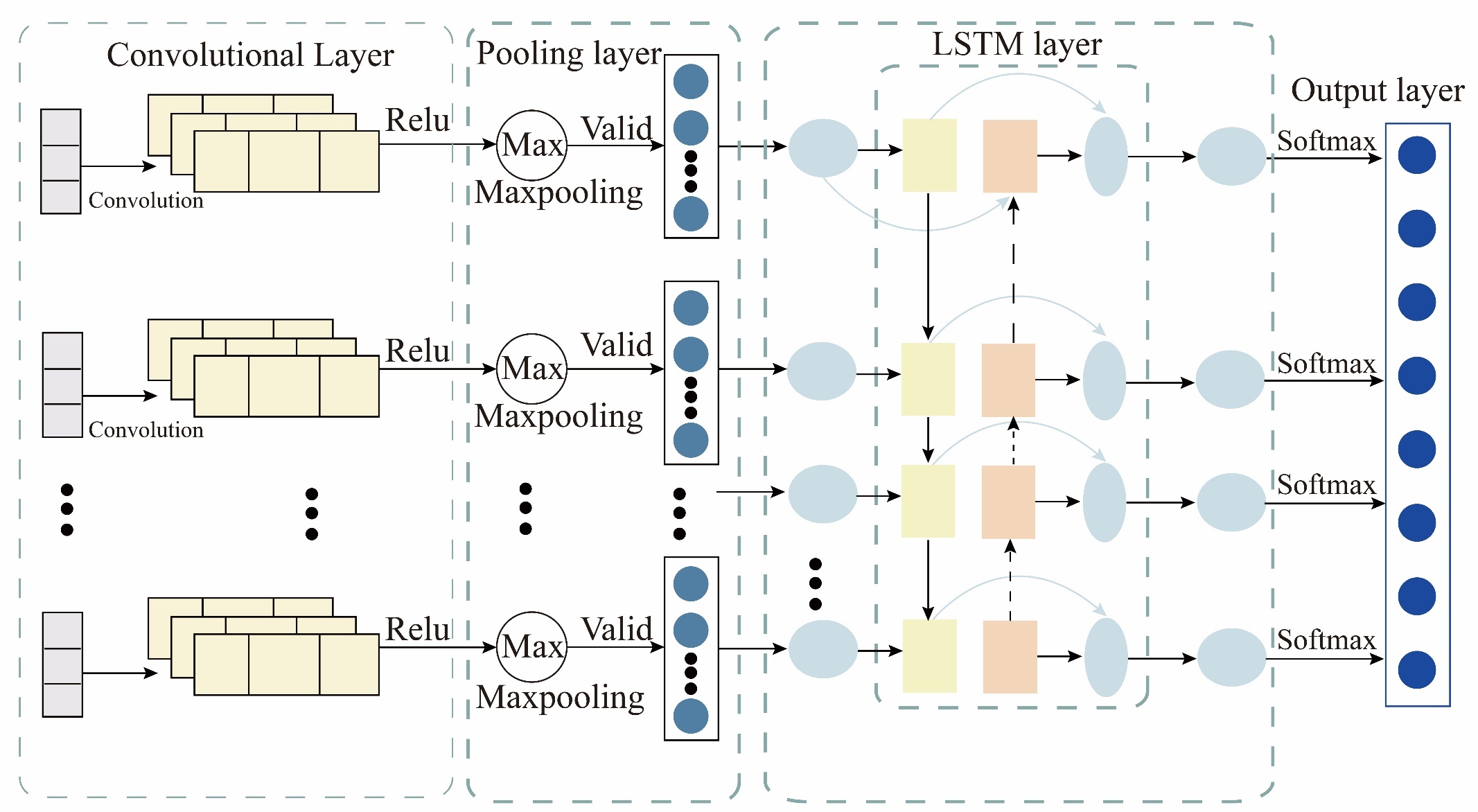
Figure A5. The framework of CNN-LSTM.

## A.3 The architecture of CNN-GRU-AM

CNN-GRU-AM is a hybrid deep learning architecture that combines CNNs with GRU and AM (Figure A6)[32]. In CNN-GRU-AM, the CNN component is responsible for capturing local contextual information and extracting important features from the input data (Figure A6). The GRU component to model the sequential dependencies and capture both forward and backward contextual information (Figure A6). This allows the model to have access to not only the past but also the future data, enhancing its understanding of the text. To further improve the model's ability to focus on relevant parts of the input, the AM is incorporated. The AM enables the model to dynamically weigh the importance of different parts of the input sequence. By combining these three components, CNN-GRU-AM benefits from the strengths of each architecture (Figure A6). The CNN captures local features, the GRU captures sequential dependencies, and the AM enhances the model's ability to focus on important information[32].

In the CNN part of this study, a design with 2 layers of one-dimensional convolutional layers was implemented. The number of convolutional kernels used were 4 and 8 respectively, and after each convolution, a corresponding max pooling operation was performed. ReLU was chosen as the activation function. After two rounds of convolution, pooling, and flattening, a one-dimensional global feature vector was obtained and used as the input for the GRU layer. The output of the GRU layer was then passed through a fully connected layer to produce the prediction results. The combination of CNN and GRU enables the comprehensive learning of spatiotemporal features in data.


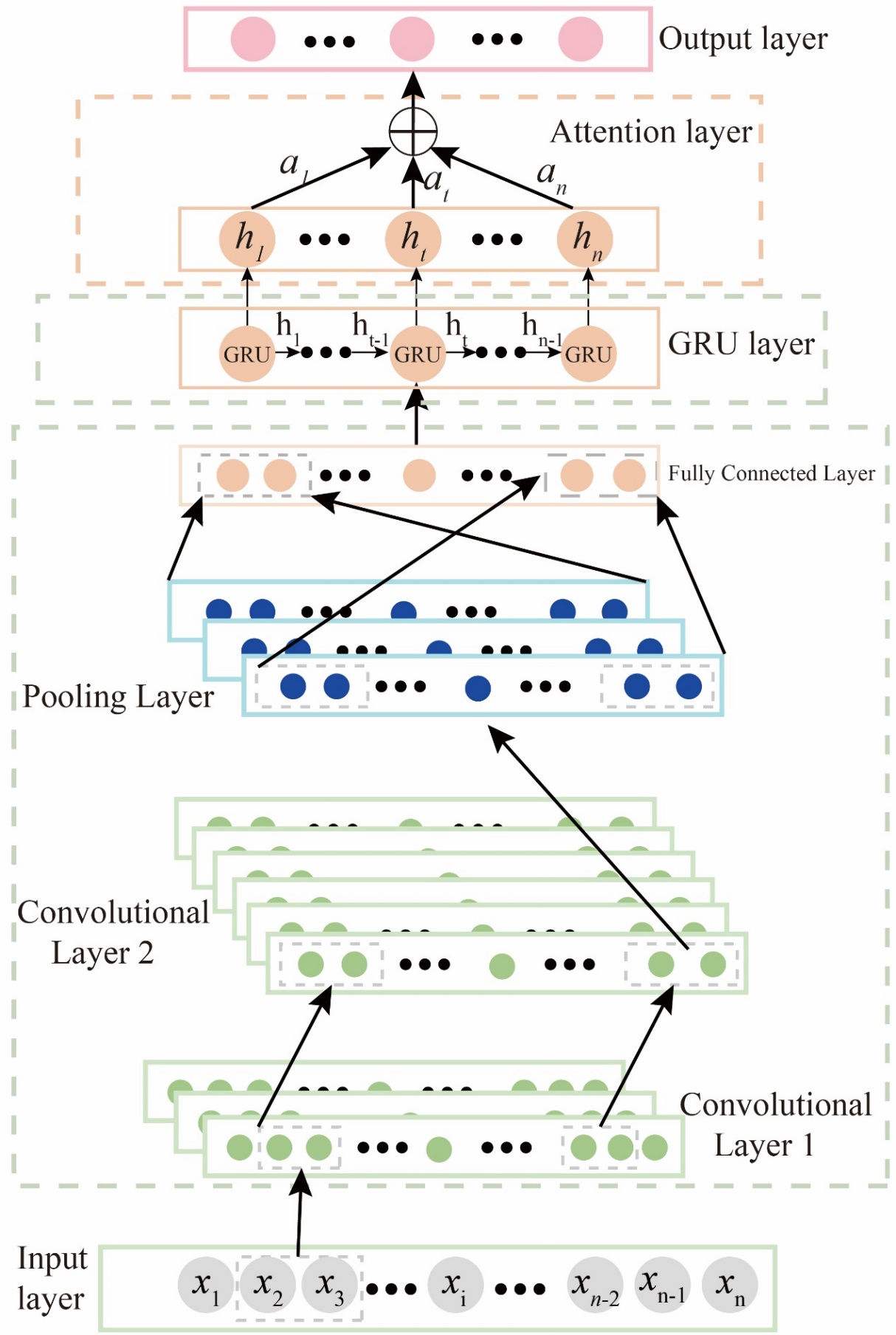


Figure A6. The framework of CNN-GRU-AM.
